# Opsin expression varies across larval development and taxa in pteriomorphian bivalves

**DOI:** 10.3389/fnins.2024.1357873

**Published:** 2024-03-18

**Authors:** Md Shazid Hasan, Kyle E. McElroy, Jorge A. Audino, Jeanne M. Serb

**Affiliations:** ^1^Department of Ecology, Evolution and Organismal Biology, Iowa State University, Ames, IA, United States; ^2^Department of Zoology, University of São Paulo, São Paulo, Brazil

**Keywords:** metamorphic competence, veliger, trochophore, GPCR, RNA-seq, Mytilidae, Ostreidae, Pectinidae

## Abstract

**Introduction:**

Many marine organisms have a biphasic life cycle that transitions between a swimming larva with a more sedentary adult form. At the end of the first phase, larvae must identify suitable sites to settle and undergo a dramatic morphological change. Environmental factors, including photic and chemical cues, appear to influence settlement, but the sensory receptors involved are largely unknown. We targeted the protein receptor, opsin, which belongs to large superfamily of transmembrane receptors that detects environmental stimuli, hormones, and neurotransmitters. While opsins are well-known for light-sensing, including vision, a growing number of studies have demonstrated light-independent functions. We therefore examined opsin expression in the Pteriomorphia, a large, diverse clade of marine bivalves, that includes commercially important species, such as oysters, mussels, and scallops.

**Methods:**

Genomic annotations combined with phylogenetic analysis show great variation of opsin abundance among pteriomorphian bivalves, including surprisingly high genomic abundance in many species that are eyeless as adults, such as mussels. Therefore, we investigated the diversity of opsin expression from the perspective of larval development. We collected opsin gene expression in four families of Pteriomorphia, across three distinct larval stages, i.e., trochophore, veliger, and pediveliger, and compared those to adult tissues.

**Results:**

We found larvae express all opsin types in these bivalves, but opsin expression patterns are largely species-specific across development. Few opsins are expressed in the adult mantle, but many are highly expressed in adult eyes. Intriguingly, opsin genes such as retinochrome, xenopsins, and Go-opsins have higher levels of expression in the later larval stages when substrates for settlement are being tested, such as the pediveliger.

**Conclusion:**

Investigating opsin gene expression during larval development provides crucial insights into their intricate interactions with the surroundings, which may shed light on how opsin receptors of these organisms respond to various environmental cues that play a pivotal role in their settlement process.

## 1 Introduction

One of the outstanding questions in marine larval biology is how do larvae detect environmental cues which initiate metamorphosis? Metamorphic competence describes the larval readiness and ability to mediate settlement on a selected surface and complete a morphogenetic transformation into the adult form (Hadfield et al., 2001; Bishop et al., 2006) and it can be divided into two parts. Settlement is a reversable behavioral phase and appears to be controlled by a dopaminergic receptor-mediated neural pathway, while metamorphosis, an irreversible morphogenetic phase, is controlled by an adrenergic receptor-mediated pathway for at least some species (Bonar et al., 1990). Environmental stimuli that influence or initiate these phases are likely hierarchical and include both physical and biochemical cues (Say and Degnan, 2020). Physical cues that drive larval behavior and may play a role in metamorphic competence include light (Bayne, 1964; Rittschof et al., 1998), surface texture, water flow, and temperature (reviewed in Bonar et al., 1990), but the morphogenetic transformation into competent larvae typically requires the identification of biochemical cues that will trigger additional changes. Some likely candidates are chemicals released by conspecific adults or are present on the substrate appear to promote larval competence by indicating the quality of the habitat (Rodriguez et al., 1993; Rittschof et al., 1998). Surprisingly, the nature of the environmental cues that trigger settlement and metamorphosis are largely unknown for most marine invertebrates, and the likelihood of species specificity adds another layer of complexity to this scenario (Zeng et al., 2022).

Marine bivalves, like many other mollusks, have free-swimming, planktonic larvae that spend a variable amount of time in the water column before settling onto the benthos. A classic example of this biphasic lifecycle is in the Pteriomorphia, a diverse clade including scallops, mussels, oysters, and pearl oysters. Despite significant differences in the duration of the pelagic period (Marshall et al., 2010), these species share very similar developmental stages with a conserved morphology (Loosanoff et al., 1966). Within hours after gastrulation, the trochophore is formed as a ciliated larva that lasts until the secretion of the larval shell (Carter et al., 2012). The second developmental stage is the veliger, marked by two valves embracing the larval body and an enlarged ciliated velum used for swimming (Waller, 1981). It is also during this larval stage that a pair of simple eyespots is formed (Cragg, 2016). The last stage is the pediveliger, remarkable for the presence of a long foot associated with crawling behavior (Cragg, 2016) and is likely used as a sensory organ during settlement (Croll et al., 1997). The pelagic phase ends when pediveligers settle onto suitable surfaces where metamorphosis will result in the benthic juvenile. As in the case for most benthic organisms, the molecular basis of larval sensory receptors is largely unknown in bivalves (Zeng et al., 2022), which raises the question of how environmental cues are perceived.

Organisms detect environmental stimuli using an array of sensory receptors, and the duplication and divergence of these receptors provide evolutionary opportunities for expansion into new ecological niches. The seven-transmembrane G-coupled protein receptor (GPCR) is the largest superfamily of transmembrane receptors that allow organisms to detect environmental stimuli, hormones, and neurotransmitters (Fredriksson et al., 2003). One of the most important sensory receptors is opsin, a GPCR present across Metazoa. Opsins bind to a chromophore molecule, typically 11-*cis* retinal, to form a photopigment capable of absorbing photons and initiating phototransduction (Terakita, 2005). Opsins are classified based on the type of photoreceptors they were discovered in (e.g., rhabdomeric “r-opsins” and ciliary “c-opsins”), the G-protein they couple with (e.g., G_q_ vs. G_t_), and phylogenetic relationship (e.g., the “tetraopsin” clade which includes retinochrome, G_o_-opsins and neuropsin; Shichida and Matsuyama, 2009; Porter et al., 2012), see also summary table for opsin function in (McElroy et al., 2023). In addition to mediating vision in animal eyes, opsins are known to be used for photoreception in extraocular tissues (Rawlinson et al., 2019; Calligaro et al., 2021) and also acting in light-independent functions, such as taste (Leung et al., 2020). Recently, we discovered extensive variation in opsin content across Mollusca, ranging from three to 63 genomic copies (McElroy et al., 2023). Among our findings was that pteriomorphian bivalves exhibit lineage-level expansions in several different types of opsins. While mantle eyes in adult animals have evolved numerous times in Pteriomorphia (Audino et al., 2020), opsin expansions are not restricted to eyed lineages (McElroy et al., 2023). From a gene expression perspective, previous RNA-seq analysis of eyes in the bay scallop *Argopecten irradians* revealed multiple duplications of the G_q_-coupled r-opsins (Porath-Krause et al., 2016), the primary visual opsin used by invertebrates, such as arthropods (Cronin and Porter, 2014), cephalopods (Hubbard and St. George, 1958), and scallops (Kojima et al., 1997). Initially, this finding raised the possibility that opsin diversification is tied to the evolution of novel, specialized photosensory structures in bivalves. Surprisingly, the extensive opsin duplication—including G_q_protein coupled r-opsins - in the mussels Mytilidae (McElroy et al., 2023), which do not have adult eyes, does not support this relationship. In addition, the data suggests that neither the presence nor the complexity of eyes is necessarily tied to an increase in opsin copy number. Such apparent contradiction raises the question of where and when such remarkable diversity of opsin copies is expressed. Consequently, we hypothesize that bivalve opsins might be expressed in different biological contexts, such as larval development and competency.

Identifying where the diverse repertoires of opsins are expressed in pteriomorphian species such as mussels, oysters, and scallops is a critical first step toward understanding the evolutionary pressures driving opsin diversification. In this context, exploring opsin expression across larval development might help elucidate how opsins are used during the pelagic lifecycle and their roles across different stages. Therefore, we expect adult and larval stages to express different opsin repertoires. More precisely, we hypothesize that: (1) opsins expressed in mantle eyes are unique to these organs; (2) opsin repertoire varies across development but not so much across phylogenetically close species; (3) the expression of some opsin types might be stage-dependent; and (4) the highest number of opsin expression occur in the pediveliger stage when larvae search for environmental clues that can indicate suitable surfaces for settlement.

To address these questions, we investigated opsins in the context of life stages to determine where and when these genes are expressed. We examined pteriomorphian species with publicly available annotated genomes from five eyeless species: the Portuguese oyster, *Crassostrea angulata*; the Pacific oyster, *Crassostrea gigas*; the Akoya pearl oyster, *Pinctada fucata*; the Korean mussel, *Mytilus coruscus*; and blue mussel, *Mytilus edulis*. We also examined two species that possess eyes as adults: the Chinese scallop, *Chlamys farreri*, and the king scallop, *Pecten maximus*. Using these seven target species, we were able to characterize changes in opsin expression across bivalve development. For each species, we leveraged available RNA-seq data for three major larval stages, i.e., trochophore, veliger, and pediveliger. We also retrieved data from specific adult tissue types, such as the adult mantle, a known photosensitive tissue (Kennedy, 1960), and adult mantle eyes (when present). By generating a robust phylogeny of pteriomorphian opsins we were able to ensure that variations in expression levels can be interpreted in the context of extensive lineage-level duplications observed in bivalves (McElroy et al., 2023). Our results reveal that opsin expression patterns across larval development are largely species-specific, although closely related species share the expression of some opsin types. Interestingly, larval and adult samples reveal significant differences in opsin repertoire. More opsins are expressed during the larval stages, with increasing opsin expression during the veliger and pediveliger stages, relative to adult tissues. By linking these data to a species’ life history, we provide the first comparative steps to understanding the biological relevance of opsin types and their evolution in marine bivalves.

## 2 Methods

### 2.1 Genomic and transcriptomic data collection

To examine changes in opsin expression across Pteriomorphia, we identified species pairs with both publicly available annotated genomes and RNA-seq data collected at three developmental stages (i.e., trochophore, veliger, pediveliger) and from adult tissues. All RNA-seq data needed to be (1) based on Illumina paired-end sequencing with (2) relatively high and similar sequence depth across studies. Seven species from four families met our criteria: mussels *Mytilus edulis* and *M. coruscus* (Mytilidae); oysters *Crassostrea gigas* and *Cr. angulata* (Ostreidae); the pearl oyster *Pinctada fucata* (Margaritidae); and scallops *Chlamys farreri* and *Pecten maximus* (Pectinidae; Supplementary Table S1). For some species (e.g., *Cr. gigas, Pi. fucata,* and *Pe. maximus*), a single study did not include both larval and adult tissues, so a second study was obtained for the larval—adult comparison. Only data from control treatments were used for our analyses. Biological replicates were available for all tissue types across focal species with the exception of *M. edulis* (larval stages), *M. coruscus* (mantle), *Pi. fucata* (all tissue), and *Ch. ferreri* (larval stages; Supplementary Table S1). All transcriptomic annotated data was retrieved from the NCBI Sequence Read Archive (SRA; Supplementary Table S1), except the *Pi. fucata* data, which was downloaded from Takeuchi et al. (2012). We used the sratoolkit v3.0.0 (Heldenbrand et al., 2017) to download the RNA-seq datasets from the NCBI SRA database and fastp v0.23.2 (Chen et al., 2018) was used to ensure quality control by eliminating low-quality reads and adapters from the downloaded FASTQ files.

### 2.2 Opsin sequence analysis and classification

McElroy et al. (2023) demonstrated extensive lineage-specific opsin expansions in Mollusca, with bivalves having highly variable opsin content. To place opsins from our focal bivalve species into proper phylogenetic context, we collected opsin sequences from 23 high-quality pteriomorphian genome assemblies (Supplementary Table S2, species bolded used for expression analysis). Building on the results of McElroy et al. (2023), we used the gene-family assembly pipeline BITACORA v1.3 (Vizueta et al., 2020), incorporating Gene Model Mapper (GeMoMa; Keilwagen et al., 2016, 2018), to *de novo* predict genes based on alignments of the same high-quality molluscan opsin protein sets. We ran the predicted genes through the Phylogenetically Informed Annotation Pipeline (PIA; Speiser et al., 2014; modified version downloaded)^1^ to identify opsins based on the Light Interacting Toolkit (LIT_1.1; r_opsin_20_rtrans.fas). We also aligned the high-quality curated reference opsin protein sequences (McElroy et al., 2023) from close relatives to additional genome assemblies (e.g., *Crassostrea gigas* for *Cr. angulata*) using miniprot v0.7 (Li, 2023) and then extracted transcripts and protein sequences for each gene using gffread v0.12.7 (Pertea and Pertea, 2020). We inspected alignments in MEGA X (Kumar et al., 2018) to combine results from these two approaches and aid in manually completing gene models (here, a complete GPCR Class A 7tm_1 domain) along with tblastn (NCBI BLAST+ v2.13.0; Camacho et al., 2009) hits in their respective genomes. All candidate opsins had a retinal-binding lysine residue homologous to K296 in bovine rhodopsin.

Recently, a closely related 7-transmembrane GPCR was identified in mollusks, annelids, and nemerteans as being more closely related to opsins than melatonin receptors and named “pseudopsins” (De Vivo et al., 2023). For outgroup sequences, we used these “pseudopsins,” along with melatonin receptors, and the opsin-like GPCRs from the placozoan *Trichoplax adhaerens* referred to as “placopsins” (XP_002113363.1, XP_002112437.1). To collect pseudospin and melatonin receptor sequences from additional species, we similarly mapped protein sequences from close relatives to the genome assemblies (e.g., *Cr. gigas* for *Cr. angulata*).

We then used mafft v7.481 (Kuraku et al., 2013) to align the opsin and outgroup amino acid sequences using the EINSI strategy (−maxiterate 1,000 –genafpair), then generated a phylogenetic tree using maximum likelihood analysis with IQ-TREE2 v2.1.3 (Minh et al., 2020) using the protein substitution model JTT + F + R9, and 1,000 ultrafast bootstrap for node support. For the purposes of visualization, we pruned the resulting tree using the R package ape v5.7.1 (Paradis et al., 2004) that only the opsin sequences from the seven species analyzed here for gene expression are present in the topology.

### 2.3 Opsin nomenclature

The opsin literature has a long list of synonymies for opsin types. Here, we use common names and the short-hand synonyms that often indicate that opsin’s G-protein signaling pathway: r-opsin = G_q_-opsin, which includes the arthropod and cephalopod visual opsins and the vertebrate melanopsin; xenopsin = G_x_-opsin, an opsin type found in lophototrochozoans; G_o_-opsin; neuropsin = Opn5; retinochrome = RTC, and peropsin.

To make orthologous gene comparisons among species and to distinguish genes resulting from paralogous duplication, we developed a nomenclature based on the phylogenetic topology of pteriomorphian opsins. Our nomenclature only applies to this study, as adding additional opsin sequences to a phylogenetic analysis could alter the placement of gene duplications that we identified. However, we think that future attempts at a comprehensive opsin nomenclature should be grounded in phylogenetics. Briefly, the first three letters of a gene name are determined by the first capital letter of the genus and the first two letters in lowercase of the species name (e.g., “Med” for *Mytilus edulis*). A period separates the abbreviated Latin binomial from the alphanumeric code identifying the opsin type (homolog), such as “xenopsin” (e.g., “opnGx”). The next part of the name is a single letter capitalized indicating the opsin clade membership if the opsin type is divided into multiple clades, for example, clades A versus B in xenopsin (e.g., “opnGx.B”). If there is a paralogous gene duplication, it is shown as an Arabic numeral with the clade letter (e.g., “opnGx.B1”). A period separates the clade membership with estimated time of when the paralogous duplication occurred. “MY” specifies a duplication along the “Mytilidae” lineage (e.g., “opnGx.B1.MY”).

### 2.4 Quantifying gene expression

Typically, bivalve larvae are sampled by hundreds or thousands of individuals per time point. Many of the studies used here had multiple pooled samples at the same time point or had two collection times within a single developmental stage, for example, 17 and 21 days post-fertilization (dpf) across the pediveliger stage. In these situations, we did a single mapping process with multiple samples and then averaged these data to get a single transcripts-per-million (TPM) value representing that developmental stage (“pediveliger”; Supplementary Table S1; e.g., *Crassostrea angulata*). We applied the same approach when there were multiple RNAseq data for adult tissues (Supplementary Table S1; e.g., *Mytilus edulis*). Another caveat with the data is that the length developmental stages can vary among species (hours to days) or within a species when influenced by environmental inputs like temperature (reviewed in Cragg, 2016). Thus, there may be changes in gene expression during a prolonged stage that were not captured when examining a single collection time point.

We combined nucleotide sequences of curated opsins for each species with their publicly available genome annotations, removing any redundancies created by the opsin sequence addition. We then used Salmon v1.9.0 (Patro et al., 2017) for pseudo-alignment-based quantification of each SRA dataset (Supplementary Table S1) and collected the transcripts-per-million (TPM) values for downstream comparisons. To account for possible noise, we then categorized opsins as expressed (present) in each sample if the TPM value was above the 10th percentile of values from each study (Supplementary Table S3).

Finally, we selected four well-established housekeeping genes to compare with opsin expression: actin (ACTB), glyceraldehyde-3-phosphate dehydrogenase (GAPDH), succinate dehydrogenase (SDHA), and polyubiquitin-C (UBC; Silver et al., 2008; Huan et al., 2016). All the housekeeping genes were extracted from the seven focal species and tblastn (NCBI BLAST+ v2.13.0; Camacho et al., 2009) was used to find the hits of these protein sequences in their respective genomes. All four housekeeping genes were recovered except UBC in *Crassostrea angulate*, GAPDH from *Mytilus coruscus*, and SDHA and UBC in *Pinctada fucata*. Finally, the transcripts-per-million (TPM) values for these housekeeping genes were extracted from the same SRA datasets from which we determined opsin expression (Salmon v1.9.0; Patro et al., 2017).

## 3 Results

### 3.1 Pteriomorphian opsin phylogeny reveals extensive protein diversity and gene duplications

**Figure 1:**
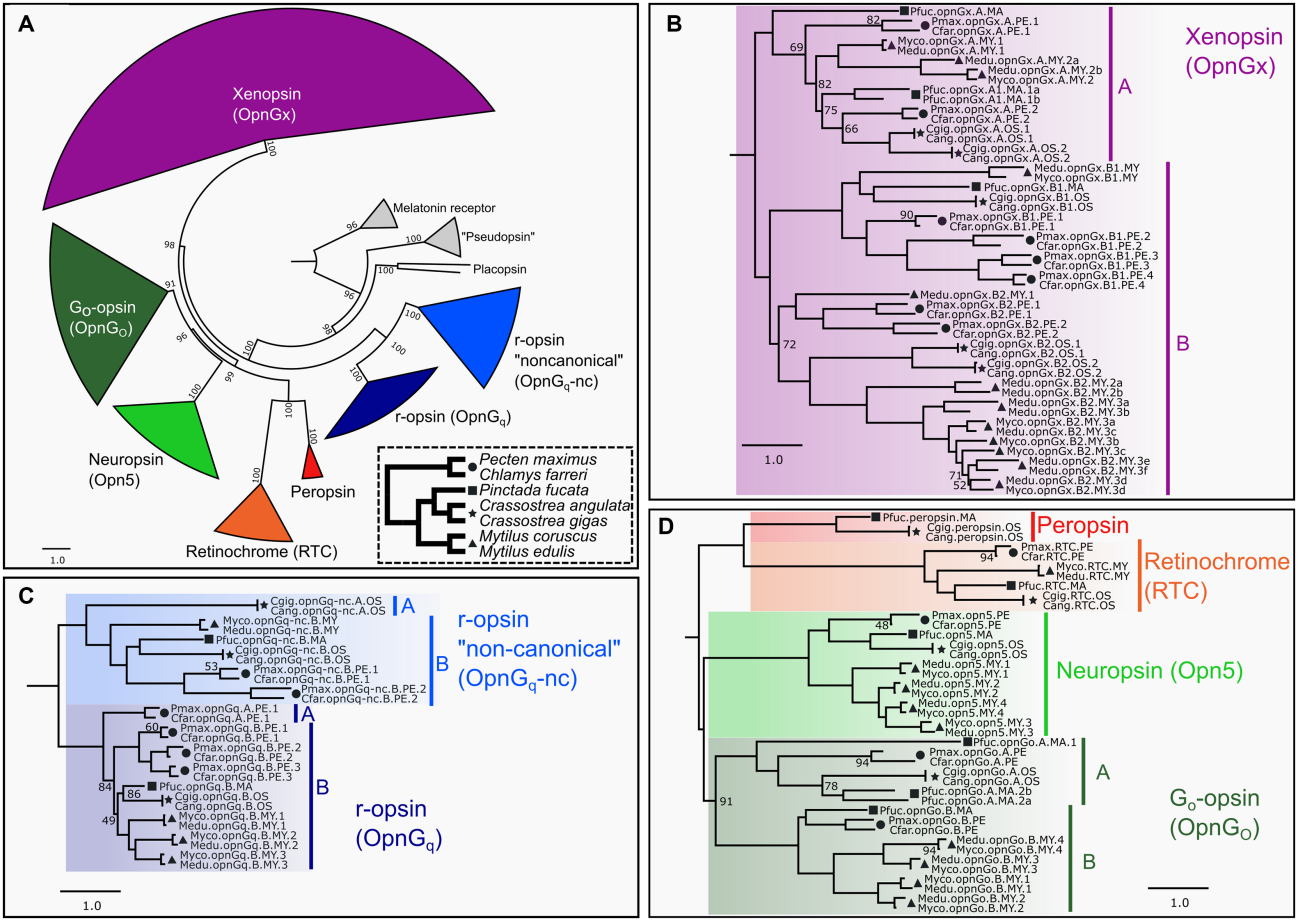
ML opsin phylogeny based on 447 pteriomorphian opsin and outgroup sequences. Color-coding of clades is by opsin type and same through panels **(A–D)**. Symbols indicate taxonomic membership by family: circle = Pectinidae, square = Margaritidae, star = Ostreidae, triangle = Mytilidae. In panels **(B–D)**, only UF-bootstrap values <95 are show at nodes. Naming system of opsins described in Methods. **(A)** Circle phylogeny of all opsin subgroups, labeled and color-coded. Numbers of above the branches represent all UF-bootstrap values. Outgroup genes in gray. Inset panel in dotted line is a species phylogeny of the seven target species. Symbols indicate taxonomic membership by family. **(B)** Pruned topology of the pteriomorphian xenopsin subgroup. Major clades A and B indicated by vertical bars. **(C)** Pruned topology of the pteriomorphian G_q_-opsin subgroup. Major clades A and B in canonical and non-canonical G_q_-opsin shown as vertical bars and indicate a gene type duplication in pre-Pteriomorphia. **(D)** Pruned topology of pteriomorphian “tetraopsins” *sensu* Ramirez et al. (2016). Major clades A and B indicating a gene type duplication in pre-Pteriomorphia highlighted by vertical bars. A full topology is provided in Supplementary Data 1.

A ML tree was generated to place opsins from target pteriomorphian species into the opsin types identified in McElroy et al. (2023). Broadly, we recapitulated previous relationships of molluscan opsin groups (Figure 1A; Supplementary Data 1) and evidenced lineage-level duplications of many opsins in this group of bivalves recently demonstrated in McElroy et al. (2023). This phylogeny provided a framework to identify putative paralogs and to estimate in which taxonomic lineages gene duplications or losses may have occurred. Ultimately, this phylogenetic framework allowed for more accurate comparations of gene expression among species.

Across Mollusca, genomes contain opsins from as many as seven distinct clades, but lack c-opsins and cnidopsins (McElroy et al., 2023). We phylogenetically classified 447 opsins sequences mined from 23 pteriomorphian genomes, including 119 from our seven focal species into the seven types of opsins: canonical or noncanonical G_q_-opsins (= r-opsins), neuropsin, G_o_-opsin, xenopsin (= G_x_-opsin), peropsin, and retinochrome (Figure 1A). All identified opsin sequences possessed a retinal-binding lysine residue homologous to K296 in the bovine rhodopsin positional naming system indicating the capacity to form a photopigment. Gene duplications were observed in xenopsin (Figure 1B), both canonical and non-canonical G_q_-opsins (Figure 1C), G_o_-opsin and neuropsin (Figure 1D). Some of these duplication events appear to be deep within the Pteriomorphia before the split of the four families examined (e.g., xenopsin clade B), while others are at the family-level, such as r-opsin paralogs in Pectinidae and Mytilidae (Figure 1C). Multiple rounds of gene duplication were estimated to occur in the xenopsin clade B for *Mytilus* and pectinid species, while *Crassostrea* has a single duplication event and *Pi. fucata* has only one gene from that xenopsin clade (Figure 1B). The xenopsin clade A appears to be less expansion-prone than xenopsin clade B, but gene duplication is evident in *Pi. fucata*, *Crassostrea*, and *Mytilus* (Figure 1B). Duplications of neuropsin were only observed in *Mytilus*, which has a lineage-level expansion resulting in four opsins vs. one in the other species examined here. As in McElroy et al. (2023), a single retinochrome was found in these pteriomorphian genomes (Figure 1D), and only *Crassostrea* and *Pi. fucata* had a copy of peropsin (Figure 1D).

### 3.2 Larval development extensively recruits different opsin types

**Figure 2:**
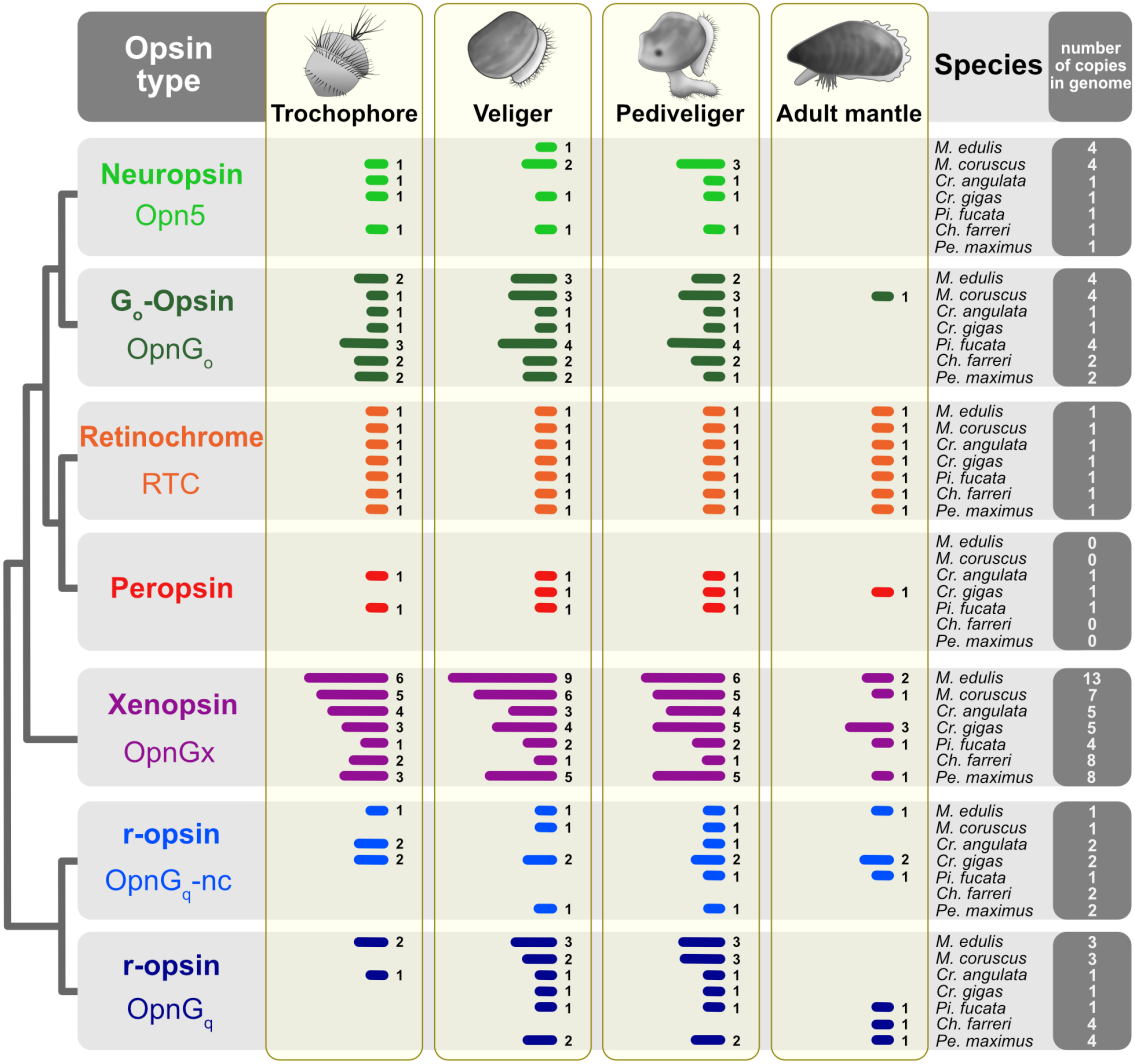
Opsin gene expression across three larval stages and the adult mantle for seven pteriomorphian species. Opsins are color-coded by type as in Figure 1. Presence of gene expression shown by bars; Arabic numerals to the right of the bars are the number of opsins in that tissue sample. Expression was treated as “off” if the TPM value was below the 10th percentile of values determined from each study. Thresholds shown in Figures 3, 4 and Supplementary Figures S1, S2. Total number of opsins by type in a given species’ genome indicated in the far-right column.

**Figure 3:**
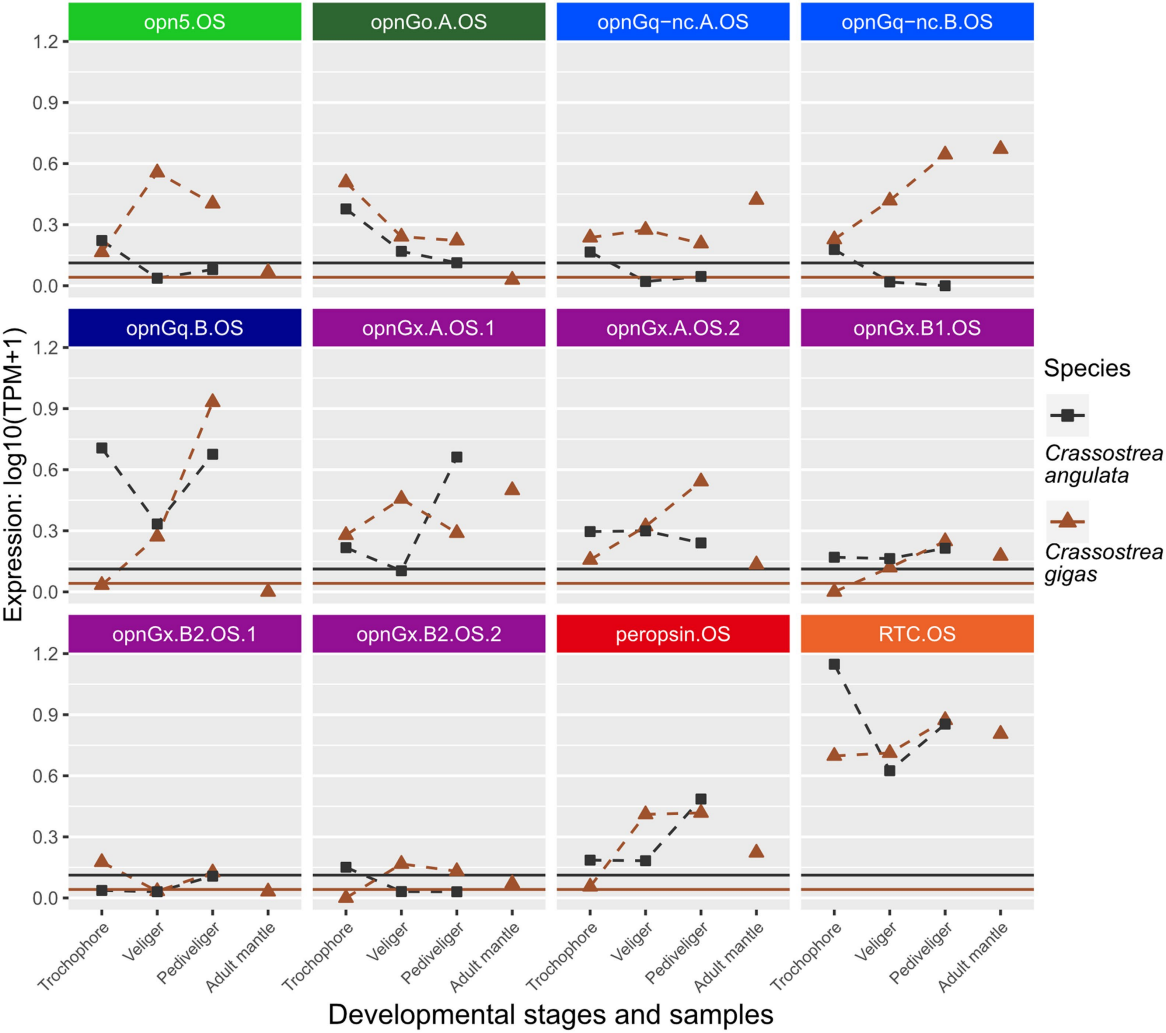
Changes in opsin gene expression across larval stages and adult tissue for two oyster species, *Cr*. *angulata* (square) and *Cr. gigas* (triangle; Ostreidae). Expression data collected in the same study are connected by dotted lines showing ontogenetic changes in expression levels within a species. Only larval data for *Cr. angulata.* Each panel is an interspecific comparison of one opsin ortholog, which are color-coded by opsin type as in Figure 1. To account for noise in the data, colored horizontal lines are the transcripts-per-million (TPM) values above the 10th percentile from each study. Opsin nomenclature described in Methods: Opn5 = neuropsin; OpnG_o_ = G_o_-opsin; OpnG_q_-nc = rhabdomeric noncanonical G_q_-opsin; OpnGq = rhabdomeric canonical G_q_-opsin; OpnG_x_ = xenopsin; RTC = retinochrome.

Some general patterns emerged from the opsin expression data (Figure 2; Supplementary Data 2). First, all opsin types are expressed across the three larval stages, trochophore, veliger, and pediveliger, in most species. Second, few opsin types are expressed in the adult mantle tissue. This was observed across all seven species. At one extreme, neuropsin (opn5) is below the threshold of expression in the adult mantle for all focal taxa and treated as “off” (Figure 2). Of the three species that have a peropsin gene (*Cr. angulata*, *Cr. gigas*, and *Pi. fucata*), expression occurs during the trochophore stage in *Cr. angulata* and *Pi. fucata*, veliger and pediveliger in all three species, but only the mantle tissue of *Cr. gigas* (summarized in Figure 2, see also Figure 3; Supplementary Figure S2). This type of pattern is also prevalent with xenopsins. For example, of the 13 xenopsin in the *M. edulis* genome which are commonly expressed during larval stages, only two copies are present in the adult mantle (summarized in Figure 2, see also Supplementary Figure S1). Third, the number of opsin genes expressed for a given type is higher in the veliger and pediveliger stages than in the trochophore for most species. To summarize, 76 opsins were expressed in the veliger stage across all focal species versus 57 and 76 genes in the trochophore and pediveliger stages, respectively (Figure 2). While not a strong trend, this pattern is notable for the xenopsin (opnG_x_) in mytilid species *M. coruscus* and *M. edulis* (Figure 2) with nine and six xenopsins being expressed during the veliger stage, respectively, versus six and five xenopsins in the pediveliger stage.

### 3.3 Pteriomorphian larvae utilize species-specific opsin repertoires

**Figure 4:**
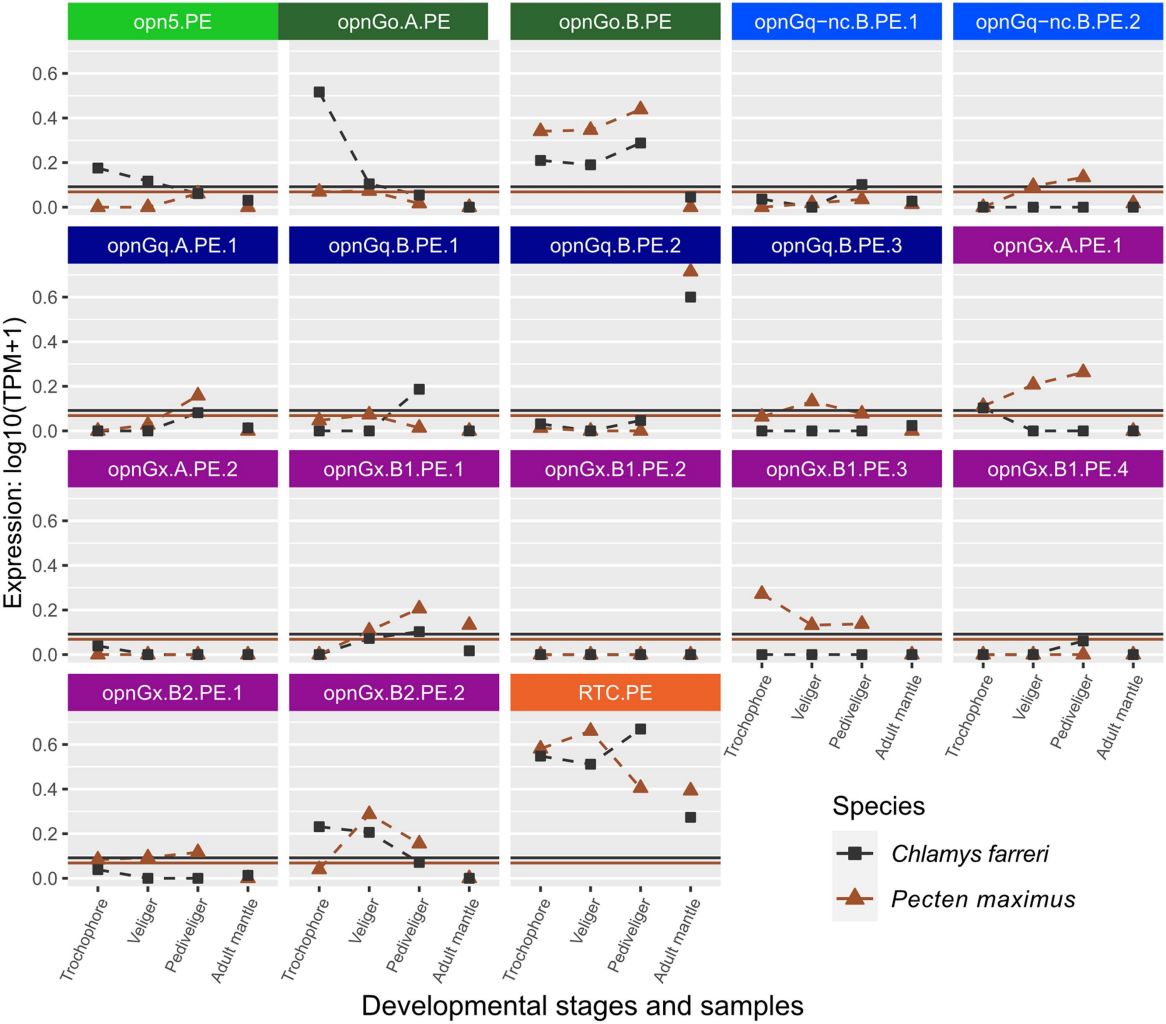
Changes in opsin gene expression across larval stages and adult tissue for two scallop species, *Ch. farreri* (square) and *Pe. maximus* (triangle; Pectinidae). Each panel is an interspecific comparison of one opsin ortholog, which are color-coded by opsin type like Figure 1. Expression data collected in the same study are connected by dotted lines showing ontogenetic changes in expression levels within a species. Larval stages did not have biological replicates for *Ch. farreri*. To account for noise in the data, colored horizontal lines are the transcripts-per-million (TPM) values above the 10th percentile from each study. Naming system for opsins described in Methods: Opn5 = neuropsin; OpnG_o_ = G_o_-opsin; OpnG_q_-nc = rhabdomeric noncanonical G_q_-opsin; OpnGq = rhabdomeric canonical G_q_-opsin; OpnG_x_ = xenopsin; RTC = retinochrome.

When looking at changes in relative expression level of specific opsins rather than presence/absence of expression, no clear patterns emerge, except that retinochrome (RTC) was the most highly expressed gene across the focal species (Figures 3, 4; Supplementary Figures S1, S2) when considering non-eye tissue samples. Instead, changes during larval development are largely lineage-specific among our seven focal species. For example, when comparing opsin expression between the two oyster species, *Cr. angulata* and *Cr. gigas*, only two of the 12 genes, a G_o_-opsin, opnGo.A.OS, and a xenopsin, opnGx.B1.OS, have similar changes (Figure 3). Seven of the opsin genes have opposing expression profiles (e.g., opn5.OS, opnGq-nc.B.OS, and opnGq.B.OS; Figure 3). Comparing the oysters to their most closely related family, Margaritidae (*Pi. fucata*), gene expression is dissimilar for the orthologous neuropsin (opn5), which is not expressed in any of the *Pi. fucata* samples (Supplementary Figure S2), and the orthologous xenopsin (opnGx.B1.MA) is below the expression threshold for the *Pi. fucata* (Supplementary Figure S2). The three remaining orthrologs, opnGq.B, peropsin, and RTC, have grossly similar expression patterns in the larvae with highest levels of expression in pediveliger (opnGq.B and peropsin) or trochophore (RTC; Supplementary Figure S2; Figure 3). The remaining *Pi. fucata* opsins cannot be directly compared to oyster opsins due to lineage-specific duplications in xenopsins, G_o_-opsins, and noncanonical r-opsins for each family (Figures 1B–D).

Opsin expression between the pair of scallop species, *Ch. farreri* and *Pe. maximus*, appears to be more conserved than in Ostreidae. When genes were above the expression threshold, expression patterns were more similar among larval stages and when those stages were compared to the adult mantle tissue (e.g., opnGo.B.PE, opnGq.B.PE.2; Figure 4). However, expression levels of many scallop opsins were low, and often only one of the species pair had expression above its species-specific threshold. For example, low expression of xenopsin (= opnG_x_) was observed for both species in genes opnGx.A.PE.2, opnGx.B1.PE.2, but only *Pe. maximus* has expression above the threshold for opnGx.B1.PE.3 and opnGx.B2.PE.1 (Figure 4).

Opsin expression between the mytilid species, *M. coruscus* and *M. edulis*, was the most conserved. Both relative level and expression pattern across all opsin types were mirrored between the species (Supplementary Figure S1). However, genomic content varied, most notably for the xenopsin type (Figure 2). *Mytilus edulis* had additional copies of xenopsin that ranged from one new copy in the A clade to five more copies in B2 clade (e.g., opnGx.A.MY.2b, opnGx.B2.MY.3e, opnGx.B2.MY.3f; Supplementary Figure S1). These copies are the result from a series of paralogous duplication events within the Mytilidae (Figure 1B).

### 3.4 Opsins are relatively more expressed in larvae than in the adult mantle margin, except for adult eyes

**Figure 5:**
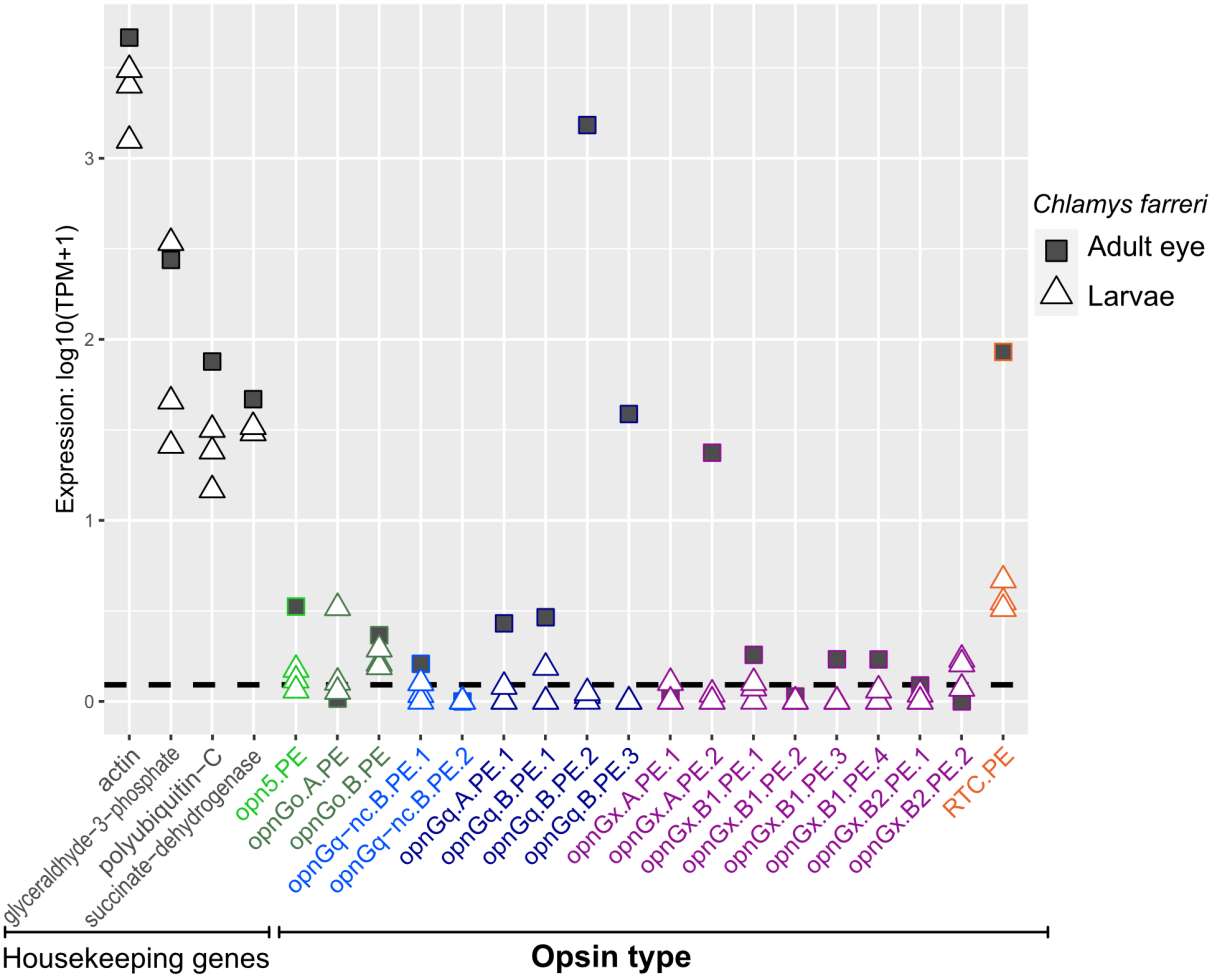
Opsin gene expression in adult eye (square) and the three larval stages (triangle) for the scallop *Ch. farreri.* Opsin type is along the x-axis and color-coded like Figure 1, following the naming system described in Methods. Relative expression levels (log transformed) on the y-axis. Four well-established housekeeping genes (left) were used to compare with opsin expression. Dotted horizontal lines are the transcripts-per-million (TPM) values above the 10th percentile from each study. All three larval stages are shown; information about a specific larval stage is in Figure 4.

Opsins were expressed at relatively lower levels in adult mantle tissue than in larvae (Figures 3, 4; Supplementary Figures S1, S2). This was observed across all opsin types in all focal species with the exceptions of two non-canonical r-opsins and one xenopsin (opnGx.A.OS.1) in *Cr. angulata* (Figure 3) and one of the pectinid-specific r-opsin paralogs, opnGq.B.PE.2, in the two scallop species (Figure 4). In contrast, when eyes were present, opsin expression was higher in eye tissue than in mantle or any larval stage. The relative expression of 12 of the 18 scallop opsins were higher in the adult eye samples of *Ch. farreri* (Figure 5). These 12 opsins represent the six opsin types that scallops possess (pectinids do not have a peropsin; Figure 2), and 10 of these opsin genes are pectinid-specific paralogs from the xenopsin A and B1 (Figure 1B) clades, non-canonical and canonical r-opsin clades (Figure 1C), and G_o_-opsin clade (Figure 1D). One copy of the paralog pairs of xenopsin (opnGx.A.PE.2), G_o_-opsin (opnGo.B.PE) and non-canonical r-opsin (opnGq-nc.B.PE.1) have relatively higher expression in eye tissue than in the larvae, while expression all four canonical r-opsin paralogs increased between 5.6 to 18.5 K fold in the eye (Figure 5). To assess whether the variation in opsin expression observed between larval stages and eyes extended throughout the system, we examined the expression levels of four housing keeping genes from the same samples. Our findings revealed consistently similar expression patterns of housekeeping genes between eyes and each larval stage, suggesting that the difference in opsin expression between larval stages and eyes are biologically meaningful and not the result of RNAseq data artifacts (Figure 5; Supplementary Data 2).

## 4 Discussion

The settlement and metamorphosis of pelagic larvae to benthic adults is an irrevocable transition that determines the survival and reproductive success of the animal. This process is orchestrated by some suite of sensory receptors that respond to physical and biochemical cues. One important physical cue is light, which in some species, influences the regulation of genes critical for settlement (Say and Degnan, 2020). The most ubiquitous photopigment is based on the opsin protein and it is known to be expressed in a variety of invertebrate larvae that exhibit phototactic behavior (Passamaneck et al., 2011; Gühmann et al., 2015; Neal et al., 2019; Döring et al., 2020). We recently discovered that mollusks, including pteriomorphian bivalves, exhibit gene expansions in many different opsin types, and these opsin expansions are not restricted to eyed species, but instead are taxon-specific and occur frequently in lineages with eyeless adults (McElroy et al., 2023). Identifying spatiotemporal expression patterns of these diverse opsin repertoires is a critical first step toward understanding function, specifically, how opsins might be utilized during the pelagic lifecycle and their roles across different developmental stages. Here, we compared opsin gene expression in four families of Pteriomorphia, across three distinct larval stages, i.e., trochophore, veliger, and pediveliger, with adult tissues known to be light-responsive. Our results show that pteriomorphian larvae have an extensive opsin repertoire. Likely, these larvae are capable of expressing multiple opsin transcripts during all three developmental stages examined, implying the existence of multiple photopigments and the possibility of multiple photoreceptor types in photosensitive regions of the trochophore (Yurchenko et al., 2018; Wollesen et al., 2019; Piovani et al., 2023) and the larval eyespots of the late veliger/early pediveliger stages.

As we hypothesized, opsin expression is more common in larval stages across all species examined than in the photosensitive mantle tissue of the adult (Figure 2). We found this trend to be strongest in xenopsin, where adult mantle tissue could have no expression or as many as three out of 13 xenopsins (opnG_x_) copies expressed (e.g., *M. edulis*, Figure 2). In contrast, the larvae had six to nine genes present in a given stage. This pattern was also seen in neuropsin (opn5), canonical and noncanonical r-opsins (opnG_q_), and G_o_-opsin.

Interestingly, while many of these opsin types were largely absent in the adult mantle, expression levels could be quite high in the eyes of the adult scallop (Figure 5). At least one paralogous copy of all six opsin types found in the scallop genome were expressed in the eye (e.g., opnGo.A.PE in larvae versus opnGo.B.PE in the eye; Figure 5). The majority of these genes were not exclusively expressed in the eye, but reveal the expression of a single gene copy between two disparate tissue types during the lifetime of the animal (e.g., opnGq.B.PE.1; Figure 5). If we assume that the presence of a retinal-binding lysine implies the formation of a photopigment and light sensing, gene sharing of these opsins between pelagic larvae and the pallial eyes of adults indicates exaptation (Gould and Vrba, 1982), a trait whose current role differs from its original function as the trait has been redeployed in a new biological context (co-option; True and Carroll, 2002) such as tissue type. When co-option does not involve gene duplication, the gene is shared between the old and new functions (Piatigorsky and Wistow, 1989). Since the pallial eye of the pectinids is a derived trait (Audino et al., 2020), the likely ancestral condition/function for these opsins is in the larvae. We hypothesize that the opsins were then co-opted for new visual processes in the adult eye, which would be neofunctionalization of that gene copy. Interesting, three of the four highest expressed opsins in the scallop eye are only expressed there (i.e., expression levels in the larvae were below the threshold): r-opsins (opnGq.B.PE.2, opnGq.B.PE.3) and one xenopsin (opnGx.B1.PE3; Figure 5). These cases may be examples of adaptation, where gene duplication occurs first, then the daughter paralogs evolve independent novel functions (True and Carroll, 2002). Our results suggest that the genetic machinery underlying the scallop pallial eye could be a combination of exaptative and adaptive processes. Future work should include studies to determine localized expression of opsin in larvae and validate opsin function. Futhermore, a macroevolutionary perspective of eye evolution will need to examine opsin expression across the life cycle of other pteriomorphian lineages with independently derived pallial eyes, such as Limidae and Arcidae (Audino et al., 2020), to determine if these morphologically distinct eyes evolved in a similar manner and utilize similar opsin repertoires.

### 4.1 Highest number of opsin genes occur in the later larval stages

We first identified and phylogenetically placed opsin genes from the genomes of seven focal species (Figure 1). Of the 119 opsin genes from our focal species, all but nine were expressed in at least one larval stage indicating that opsins were important to general larval function. When an opsin copy was not expressed in the larvae, these genes were almost always paralogous duplicates for that taxon or family (except for neuropsin in *Pi. fucata*; Supplementary Figure S2), suggesting that paralogs have diverged in function after duplication. Presence of opsin expression varied across developmental stages and species, but the greatest number of opsins was expressed in the two later larval stages veliger and pediveliger (76 and 76 out of 119 genes, respectively) versus 57 opsin genes in the trochophore stage (Figure 2). The only other bivalve study to examine opsin expression in larvae is from a non-pteriomorphian and eyeless species, the razor clam *Sinonovacula constricta* (Infraclass: Heteroconchia; Kong et al., 2023). Like our results, the majority of *S. constricta* opsins (17 out of 23 genes) was expressed in the larvae. Both number of opsins expressed, and relative expression levels increased from the trochophore to pediveliger stage (Kong et al., 2023). Also, like our findings (except in scallop eyes), opsins were generally lowly expressed in the adult tissues. These results provide an independent data point of opsin expression coinciding with the timing of metamorphic competence and support our hypothesis that opsins play a role in identifying the cues involved in settlement.

### 4.2 Photoisomerases retinochrome and peropsin expressed in all pteriomorphian life stages

One of the few opsins with consistent expression patterns across different species and developmental stages was retinochrome (Figure 2), which was often the most highly expressed opsin in these datasets (Figures 3–5; Supplementary Figures S1, S2). This opsin, first discovered in cephalopods (Hara and Hara, 1965; Hara et al., 1967), acts primarily as a photoisomerase for converting all-*trans* to 11-*cis* retinal (reviewed in Terakita and Nagata, 2014; Vöcking et al., 2022). That is, it likely does not drive phototransduction and instead acts to resupply 11-*cis* retinal for rhodopsin (Vöcking et al., 2021; Kong et al., 2023). Retinochrome is found across all mollusk clades (e.g., Ramirez et al., 2016; McElroy et al., 2023) and in other lophotrochozoans, though its function is only known from mollusks (Vöcking et al., 2021). Unlike other groups of opsins, retinochrome does not regularly duplicate and diversify; it is almost typically represented by a single gene in mollusks, indicating that it is likely functionally restricted (though see examples in Kong et al., 2023; McElroy et al., 2023). As in McElroy et al. (2023), no duplications of retinochrome were seen in pteriomorphian bivalves in this study. In addition to resupplying 11-*cis* retinal, retinochrome has been hypothesized to act as a storage protein for retinal (Ozaki et al., 1983). These critical functions may drive demand for retinochrome presence in all light-responsive cells, but currently little is known about opsin expression across development and tissue types in a broad range of mollusks.

The other opsin type in mollusks expected to act as an isomerase is the molluscan peropsin (Ramirez et al., 2016; Vöcking et al., 2021). Like retinochrome, this opsin is largely resistant to duplications, but has been lost numerous times (McElroy et al., 2023). Here, the two *Crassostrea* species and *Pinctada fucata* are the only taxa whose genomes encode peropsin. In both groups of species, we found peropsin expressed across all larval stages with apparently increasing expression levels from trochophore through pediveliger (Figure 3; Supplementary Figure S2). Determining if peropsin functions similarly to retinochrome in the classic molluscan visual cycle (Terakita et al., 1989) and whether it can drive phototransduction are important first steps in defining the role for this opsin. Furthermore, in species with both retinochrome and peropsins, visual (e.g., immunohistochemistry) or transcriptomic (e.g., single-cell RNA-seq) analysis should be conducted to determine if photoreceptors and other cell types express both opsins. Together, these investigations should help shed light on why some lineages maintain these putative photoisomerases, while other species lose it.

### 4.3 Increased number and expression levels of opsin in later larval stages

Opsin may have a role in larval exploration of suitable settlement sites. We found relatively higher levels of opsin expression in the veliger and pediveliger larval stages for peropsin and some of the paralogs of G_o_-opsin, canonical and noncanonical r-opsins (opnG_q_), and xenopsin (opnG_x_). Increased number and expression levels of opsins in these later stages may be related to increasing sensory needs as the larva approaches metamorphic competency. It has been demonstrated that larvae alter their response to light at different developmental stages, going from positive phototaxis in veligers to negative phototaxis in pediveligers (e.g., *Mytilus edulis* in Bayne, 1964). This likely is opsin-based, as opsin has been shown to be expressed in the larval eyespots of other marine invertebrates [Polyplacophora (Vöcking et al., 2015); *Platyneresis dumerilli* (Randel et al., 2013); the flatworm *Maritigrella crozieri* (Rawlinson et al., 2019)]. While the specific location of where each opsin expressed in pteriomorphian larvae is still unknown, the photosensitive eyespots are ubiquitous among molluscan larvae, forming in the late veliger or early pediveliger stages of bivalves (reviewed in Cragg, 2016). These simple organs located in the anterior aspect of each gill bar consist of two cells, a photoreceptor cell and a pigment cell, and can sense direction and intensity of light, but lack spatial vision (Hodgson and Burke, 1988). Both “visual” opsins, those expressed in adult image-forming eyes (e.g., G_q_-opsins in Randel et al., 2013; Vöcking et al., 2015), as well as opsins that have not been demonstrated to have a role in vision (e.g., xenopsins in Rawlinson et al., 2019), have been shown to be expressed in larval eyespots.

In pteriomorphians, opsin may play an important role in coordinating with a yet-to-be-determined chemosensory system to orchestrate larval settlement, perhaps analogous to the cryptochrome-based photosensing system in the sponge, *Amphimedon queenslandica* (Say and Degnan, 2020). In the sponge, detecting the cessation of light is required for the larvae to respond to a highly inductive biochemical cue, otherwise, larvae are unable to settle if maintained in constant light. Light was shown to influence expression of nearly 180 genes critical for settlement (Say and Degnan, 2020). Many of these genes possessed known G-protein regulatory motifs that repress the GPCR signaling of chemotransduction in *A. queenslandica* and likely maintain larvae in a state that is unable to respond to biochemical cues until larvae transition in to the dark (Say and Degnan, 2020). Future work in Pteriomorphia should examine these light-mediated changes to gene expression profiles during settlement and metamorphosis.

### 4.4 Larval opsins and light-independent functions

Another critical sensory modality in metamorphic competency is chemoreception. For many diverse marine invertebrates, GPCRs, the same superfamily as opsin, are the chemoreceptors that regulate settlement. This has been demonstrated across diverse metazoans such as the gastropod *Haliotis rufescens* (Trapido-Rosenthal and Morse, 1986), the echinoderm *Stronglylocentrotus purpuratus* (Amador-Cano et al., 2006), the sponge *Amphimedon queenslandica* (Say and Degnan, 2020), and cnidarians *Hydractinia echinata* (Schneider and Leitz, 1994) and *Acropora millepora* (Strader et al., 2018), but see (Holm et al., 1998; Tran and Hadfield, 2012). Intriguingly, Baxter and Morse (1992) proposed that the chemosensor that induces settlement and metamorphosis in the gastropod *Haliotis* is not only a GPCRs, but likely is a member of the rhodopsin-like class of GPCRs, as is opsin, which comprises subfamily A16. Perhaps some portion of the large and diverse opsin repertoire in pteriomorphian larvae function as chemoreceptors?

There is a growing body of evidence that opsins have multimodal functionality (Feuda et al., 2022). Opsin has been shown to have light-independent sensory modalities including chemosensory (Leung et al., 2020), auditory (Senthilan et al., 2012), mechanoreception (Katana et al., 2019), and temperature reception (Shen et al., 2011; reviewed in Leung and Montell, 2017). A promising candidate is xenopsin. A recently described opsin type (Ramirez et al., 2016), xenopsin is an under-characterized opsin restricted to Lophotrochozoa (Ramirez et al., 2016; Vöcking et al., 2017). It is associated with ciliary photoreceptors and may be co-expressed with G_q_-opsins (Vöcking et al., 2017; Döring et al., 2020). Xenopsin is particularly prone to large gene family expansions in both pteriomorphian and non-pteriomorphian bivalves (Figure 1; McElroy et al., 2023). Furthermore, these gene copies are most commonly expressed in the later developmental stages of pteriomorphian (summarized in Figure 2) and heteroconchian *S. constricta* larvae, with few expressed in adult tissue (Figure 3; Kong et al., 2023). For these reasons, we think xenopsins may be important for species-specific cues in development. Future work should target specific spatiotemporal expression patterns for xenopsins in bivalves across life stages.

Opsins are worthwhile proteins to explore in the context of life-stage triggers and decisions of settling in mollusks, which require multisensory inputs. Future work should be to test functions. A first step is to determine whether candidate opsins form photosensitive pigments when provided an appropriate chromophore. Assays to test if an opsin can form a functioning photopigment can be conducted in heterologous expression systems, where opsin is expressed outside of the animal and then and then absorbance spectra can be quantified (Faggionato and Serb, 2017; Smedley et al., 2022). Second, we can test whether the candidate opsin can perform as a chemoreceptor. Because GPCRs are one of the most common pharmaceutical targets (Sriram and Insel, 2018), there are high-resolution GPCR structures in dedicated repositories such as GPCRdb (Pándy-Szekeres et al., 2018) and GPCR-EXP (Chan and Zhang, 2020) available to investigate the molecular basis of GPCR structure–function relationship and characteristic features of ligand binding (reviewed in Venkatakrishnan et al., 2013). Furthermore, there are a wealth of protein ligand interaction databases that consists of a list of active site residues of a protein and the physio-chemical properties of ligands. Ligand compatibility can be examined with computational approaches allow modeling of ligand docking (e.g., GPCR-ModSim Esguerra et al., 2016) and ligand predictions based on protein models [pdCSM-GPCR (Velloso et al., 2021); others listed in Allen and Roth, 2011], such as the AlphaFill algorithm applied to Alphafold models (Hekkelman et al., 2023). These *in silco* studies could be followed up with *in vitro* testing of ligand binding to test for light-independent functions in an opsin (reviewed in Allen and Roth, 2011).

## 5 Conclusion and future directions

As larval development and metamorphosis involve dramatic morphological changes, gene expression is a crucial aspect to understand those processes in a functional framework. Here, we profiled opsin transcription across larval development in seven species of pteriomorphian bivalves, representing four distinct taxonomic families: Margaritidae (pearl oyster), Mytilidae (mussels), Ostreidae (oysters), and Pectinidae (scallops). Broadly, our results suggest that more opsins are expressed in larval than adult stages. Opsin evolution in Pteriomorphia is dynamic and lineage-level gene expansions have resulted in species from different families having very different opsin repertoires. We see that opsin expression patterns are more similar between closely related species and highly divergent across deeper evolutionary distances, except for retinochrome, which appears constitutively and highly expressed across development in all taxa. Interestingly, unlike the other five species, the scallop results indicate little to no expression of the G_q_-coupled r-opsin during larval stages, instead expressing these opsins—typically used for invertebrate vision—in adult eyes. These results point toward a scenario where opsins specialize to function in eyes. Important future research includes RNA-seq analysis and protein staining to confirm that lowly expressed opsins are indeed transcribed in larval development (Sadier et al., 2018). Additionally, a powerful setting to explore whether the evolution of opsin use in larvae vs. adult eyes has occurred in a similar or different manner among pteriomorphian bivalves would be an examination of the Arcidae (ark clams) and Limidae (file clams), as these lineages have eye types analogous to scallops (Audino et al., 2020). Last, while characterizing photopigment-forming potential, opsins also should be scrutinized for potential light-independent modalities such as ligand binding, which can be predicted bioinformatically. Overall, opsin expression in bivalve larvae is surprisingly diverse and might represent a key aspect related to perceiving environmental cues.

## Data availability statement

The original contributions presented in the study are included in the article/Supplementary material, further inquiries can be directed to the corresponding author. Code used in the data analysis can be found at https://github.com/kemcelroy/LarvaeRNAseq.

## Author contributions

MH: Data curation, Formal analysis, Investigation, Visualization, Writing – original draft, Writing – review & editing. KM: Conceptualization, Data curation, Formal analysis, Investigation, Visualization, Writing – original draft, Writing – review & editing. JA: Data curation, Investigation, Visualization, Writing – original draft, Writing – review & editing. JS: Conceptualization, Funding acquisition, Project administration, Resources, Supervision, Writing – original draft, Writing – review & editing.

## Footnotes

^1^ https://github.com/MartinGuehmann/PIA2

## References

[ref1] Allen J. A. Roth B. L. (2011). Strategies to discover unexpected targets for drugs active at G protein-coupled receptors. Annu. Rev. Pharmacol. Toxicol. 51, 117–144. doi: 10.1146/annurev-pharmtox-010510-100553, PMID: 20868273

[ref2] Amador-Cano G. Carpizo-Ituarte E. J. Cristino-Jorge D. (2006). Role of protein kinase C, G-protein coupled receptors, and calcium flux during metamorphosis of the sea urchin Strongylocentrotus purpuratus. Biol. Bull. 210, 121–131. doi: 10.2307/4134601, PMID: 16641517

[ref3] Audino J. A. Serb J. M. Marian J. E. A. R. (2020). Hard to get, easy to lose: evolution of mantle photoreceptor organs in bivalves (Bivalvia, Pteriomorphia). Evolution 74, 2105–2120. doi: 10.1111/evo.14050, PMID: 32716056

[ref4] Baxter G. T. Morse D. E. (1992). Cilia from abalone larvae contain a receptor-dependent G protein transduction system similar to that in mammals. Biol. Bull. 183, 147–154. doi: 10.2307/1542416 29304580

[ref5] Bayne B. L. (1964). The responses of the larvae of *Mytilus edulis* L. to light and to gravity. Oikos 15, 162–174. doi: 10.2307/3564753

[ref6] Bishop C. D. Huggett M. J. Heyland A. Hodin J. Brandhorst B. P. (2006). Interspecific variation in metamorphic competence in marine invertebrates: the significance for comparative investigations into the timing of metamorphosis. Integr. Comp. Biol. 46, 662–682. doi: 10.1093/icb/icl043, PMID: 21672777

[ref7] Bonar D. B. Coon S. L. Walch M. Weiner R. M. Fitt W. (1990). Control of oyster settlement and metamorphosis by endogenous and exogenous chemical cues. Bull. Mar. Sci. 46, 484–498.

[ref8] Calligaro H. Dkhissi-Benyahya O. Panda S. (2021). Ocular and extraocular roles of neuropsin in vertebrates. Trends Neurosci. 45, 200–211. doi: 10.1016/j.tins.2021.11.008, PMID: 34952723 PMC8854378

[ref9] Camacho C. Coulouris G. Avagyan V. Ma N. Papadopoulos J. Bealer K.. (2009). BLAST+: architecture and applications. BMC Bioinfo. 10:421. doi: 10.1186/1471-2105-10-421, PMID: 20003500 PMC2803857

[ref10] Carter J. G. Harries P. J. Malchus N. Sartori A. F. Anderson L. C. Bieler R. (2012). Illustrated glossary of the Bivalvia: treatise online, part N, Revised, Chapter 31, vol 1. Kansas University Paleontological Institute, Lawrence. 1–209.

[ref11] Chan W. K. B. Zhang Y. (2020). Virtual screening of human class-a GPCRs using ligand profiles build on multiple ligand-receptor interactions. J. Mol. Biol. 432, 4872–4890. doi: 10.1016/j.jmb.2020.07.003, PMID: 32652079 PMC7415681

[ref12] Chen S. Zhou Y. Chen Y. Gu J. (2018). Fastp: an ultra-fast all-in-one FASTQ preprocessor. Bioinformatics 34, i884–i890. doi: 10.1093/bioinformatics/bty560, PMID: 30423086 PMC6129281

[ref13] Cragg S. M. (2016). “Biology and ecology of scallop larvae” in Scallops: Biology, ecology and aquaculture. eds. Shumway S. E. Parsons G. J. (Netherlands: Elseiver), 31–83.

[ref14] Croll R. P. Jackson D. J. Voronezhskaya E. E. (1997). Catecholamine-containing cellls in larval and postlarval bivalve molluscs. Biol. Bull. 193, 116–124. doi: 10.2307/1542757 28575609

[ref15] Cronin T. W. Porter M. L. (2014). “The evolution of invertebrate photopigments and photoreceptors” in Evolution of visual and non-visual pigments. eds. Hunt D. M. Hankins M. W. Collin S. P. Marshall N. J. (New York: Springer International Publishing), 105–135.

[ref16] De Vivo G. Crocetta F. Ferretti M. Feuda R. D’Aniello S. (2023). Duplication and losses of opsin genes in Lophotrochozoan evolution. Mol. Biol. Evol. 40:p.msad066. doi: 10.1093/molbev/msad066, PMID: 36947081 PMC10097855

[ref17] Döring C. C. Kumar S. Tumu S. C. Kourtesis I. Hausen H. (2020). The visual pigment xenopsin is widespread in protostome eyes and impacts the view on eye evolution. eLife 9, 1–23. doi: 10.7554/ELIFE.55193, PMID: 32880369 PMC7529461

[ref18] Esguerra M. Siretskiy A. Bello X. Sallander J. Gutiérrez-De-Terán H. (2016). GPCR-ModSim: a comprehensive web based solution for modeling G-protein coupled receptors. Nucleic Acids Res. 44, W455–W462. doi: 10.1093/NAR/GKW403, PMID: 27166369 PMC4987938

[ref19] Faggionato D. Serb J. M. (2017). Strategy to identify and test putative light-sensitive non-opsin G-protein-coupled receptors: a case study. Biol. Bull. 233, 70–82. doi: 10.1086/694842, PMID: 29182499

[ref20] Feuda R. Menon A. K. Göpfert M. C. (2022). Rethinking opsins. Mol. Biol. Evol. 39:p.msac033. doi: 10.1093/molbev/msac033, PMID: 35143663 PMC8892948

[ref21] Fredriksson R. Lagerström M. C. Lundin L.-G. Schiöth H. B. (2003). The G-protein-coupled receptors in the human genome form five main families. Phylogenetic analysis, paralogon groups, and fingerprints. Mol. Pharmacol. 63, 1256–1272. doi: 10.1124/mol.63.6.1256, PMID: 12761335

[ref22] Gould S. J. Vrba E. S. (1982). Exaptation - a missing term in the science of form. Paleobiology 8, 4–15. doi: 10.1017/S0094837300004310

[ref23] Gühmann M. Jia H. Randel N. Michiels N. K. Bezares-caldero L. A. Gu M.. (2015). Spectral tuning of phototaxis by a go-opsin in the rhabdomeric eyes of Platynereis. Curr. Biol. 25, 2265–2271. doi: 10.1016/j.cub.2015.07.017, PMID: 26255845

[ref24] Hadfield M. G. Carpizo-Ituarte E. J. Carmen K. Del Nedved B. T. (2001). Metamorphic competence, a major adaptive convergence in marine invertebrate larvae. Am. Zool. 41, 1123–1131. doi: 10.1093/icb/41.5.1123

[ref25] Hara T. Hara R. (1965). New photosensitive pigment found in the retina of the squid Ommastrephes. Nature 206, 1331–1334. doi: 10.1038/2061331a0, PMID: 5838244

[ref26] Hara T. Hara R. Takeuchi J. (1967). Vision in the octopus and squid. Nature 214, 572–573. doi: 10.1038/214572a0, PMID: 6036170

[ref27] Hekkelman M. L. de Vries I. Joosten R. P. Perrakis A. (2023). AlphaFill: enriching AlphaFold models with ligands and cofactors. Nat. Methods 20, 205–213. doi: 10.1038/s41592-022-01685-y, PMID: 36424442 PMC9911346

[ref28] Heldenbrand J. Ren Y. Asmann Y. Mainzer L. S. (2017). Step-by-step guide for downloading very large datasets to a supercomputer using the SRA toolkit. protocols.io. doi: 10.17504/protocols.io.kb6csre

[ref29] Hodgson C. A. Burke R. D. (1988). Development and larval morphology of the spiny scallop, *Chlamys hastata*. Biol. Bull. 174, 303–318. doi: 10.2307/1541956

[ref30] Holm E. R. Nedved B. T. Carpizo-Ituarte E. Hadfield M. G. (1998). Metamorphic-signal transduction in Hydroides elegans (Polychaeta: Serpulidae) is not mediated by a G protein. Biol. Bull. 195, 21–29. doi: 10.2307/1542772, PMID: 28570195

[ref31] Huan P. Wang H. Liu B. (2016). Assessment of housekeeping genes as internal references in quantitative expression analysis during early development of oyster. Genes Genet. Syst. 91, 257–265. doi: 10.1266/ggs.16-00007, PMID: 27582049

[ref32] Hubbard R. St George R. C. C. (1958). The rhodopsin system of the squid. J. Gen. Physiol. 41, 501–528. doi: 10.1085/jgp.41.3.501, PMID: 13491819 PMC2194838

[ref33] Katana R. Guan C. Zanini D. Larsen M. E. Giraldo D. Geurten B. R. H.. (2019). Chromophore-independent roles of opsin apoproteins in Drosophila mechanoreceptors. Curr. Biol. 29, 2961–2969.e4. doi: 10.1016/j.cub.2019.07.036, PMID: 31447373

[ref34] Keilwagen J. Hartung F. Paulini M. Twardziok S. O. Grau J. (2018). Combining RNA-seq data and homology-based gene prediction for plants, animals and fungi. BMC Bioinfo. 19:189. doi: 10.1186/s12859-018-2203-5, PMID: 29843602 PMC5975413

[ref35] Keilwagen J. Wenk M. Erickson J. L. Schattat M. H. Grau J. Hartung F. (2016). Using intron position conservation for homology-based gene prediction. Nucleic Acids Res. 44:e89. doi: 10.1093/nar/gkw092, PMID: 26893356 PMC4872089

[ref36] Kennedy D. (1960). Neural photoreception in a lamellibranch mollusc. J. Gen. Physiol. 44, 277–299. doi: 10.1085/jgp.44.2.277, PMID: 13752503 PMC2195090

[ref37] Kojima D. Terakita A. Ishikawa T. Tsukahara Y. Maeda A. Shichida Y. (1997). A novel Go-mediated phototransduction cascade in scallop visual cells. J. Biol. Chem. 272, 22979–22982. 9287291 10.1074/jbc.272.37.22979

[ref38] Kong F. Zhaoshou R. Zhang M. Liao K. Chen D. Yan X.. (2023). Eyeless razor clam Sinonovacula constricta discriminates light spectra through opsins to guide Ca2+ and cAMP signaling pathways. J. Biol. Chem. 49:103297. doi: 10.1016/j.csite.2023.103297 PMC10788561 38043801

[ref39] Kumar S. Stecher G. Li M. Knyaz C. Tamura K. (2018). MEGA X: molecular evolutionary genetics analysis across computing platforms. Mol. Biol. Evol. 35, 1547–1549. doi: 10.1093/molbev/msy096, PMID: 29722887 PMC5967553

[ref40] Kuraku S. Zmasek C. M. Nishimura O. Katoh K. (2013). aLeaves facilitates on-demand exploration of metazoan gene family trees on MAFFT sequence alignment server with enhanced interactivity. Nucleic Acids Res. 41, W22–W28. doi: 10.1093/nar/gkt389, PMID: 23677614 PMC3692103

[ref41] Leung N. Y. Montell C. (2017). Unconventional roles of opsins. Annu. Rev. Cell Dev. Biol. 33, 241–264. doi: 10.1146/annurev-cellbio-100616-060432, PMID: 28598695 PMC5963513

[ref42] Leung N. Y. Thakur D. P. Gurav A. S. Kim S. H. Di Pizio A. Niv M. Y.. (2020). Functions of opsins in Drosophila taste. Curr. Biol. 30, 1367–1379.e6. doi: 10.1016/j.cub.2020.01.068, PMID: 32243853 PMC7252503

[ref43] Li H. (2023). Protein-to-genome alignment with miniprot. Bioinformatics 39:p.btad014. doi: 10.1093/bioinformatics/btad014, PMID: 36648328 PMC9869432

[ref44] Loosanoff V. L. Davis H. C. Chanley P. E. (1966). Dimensions and shapes of larvae of some marine bivalve mollusks. Malacologia 4, 351–435.

[ref45] Marshall R. McKinley S. Pearce C. M. (2010). Effects of nutrition on larval growth and survival in bivalves. Rev. Aquac. 2, 33–55. doi: 10.1111/j.1753-5131.2010.01022.x

[ref46] McElroy K. E. Audino J. A. Serb J. M. (2023). Molluscan genomes reveal extensive differences in photopigment evolution across the phylum. Mol. Biol. Evol. 40:p.msad263. doi: 10.1093/molbev/msad263, PMID: 38039155 PMC10733189

[ref47] Minh B. Q. Schmidt H. A. Chernomor O. Schrempf D. Woodhams M. D. von Haeseler A.. (2020). IQ-TREE 2: new models and efficient methods for phylogenetic inference in the genomic era. Mol. Biol. Evol. 37, 1530–1534. doi: 10.1093/molbev/msaa015 32011700 PMC7182206

[ref48] Neal S. De Jong D. M. Seaver E. C. (2019). CRISPR/CAS9 mutagenesis of a single r-opsin gene blocks phototaxis in a marine larva. Proc. R. Soc. B Biol. Sci. 286:20182491. doi: 10.1098/rspb.2018.2491, PMID: 31161907 PMC6571462

[ref49] Ozaki K. Hara R. Hara T. Kakitani T. (1983). Squid retinochrome. Configurational changes of the retinal chromophore. Biophys. J. 44, 127–137. doi: 10.1016/S0006-3495(83)84285-4, PMID: 6626676 PMC1434814

[ref50] Pándy-Szekeres G. Munk C. Tsonkov T. M. Mordalski S. Harpsøe K. Hauser A. S.. (2018). GPCRdb in 2018: adding GPCR structure models and ligands. Nucleic Acids Res. 46, D440–D446. doi: 10.1093/nar/gkx1109, PMID: 29155946 PMC5753179

[ref51] Paradis E. Claude J. Strimmer K. (2004). APE: analyses of Phylogenetics and evolution in R language. Bioinformatics 20, 289–290. doi: 10.1093/bioinformatics/btg412, PMID: 14734327

[ref52] Passamaneck Y. J. Furchheim N. Hejnol A. Martindale M. Q. Luter C. Lüter C. (2011). Ciliary photoreceptors in the cerebral eyes of a protostome larva. EvoDevo 2:6. doi: 10.1186/2041-9139-2-6, PMID: 21362157 PMC3062599

[ref53] Patro R. Duggal G. Love M. I. Irizarry R. A. Kingsford C. (2017). Salmon provides fast and bias-aware quantification of transcript expression. Nat. Methods 14, 417–419. doi: 10.1038/nmeth.4197, PMID: 28263959 PMC5600148

[ref54] Pertea G. Pertea M. (2020). GFF utilities: GffRead and GffCompare. F1000Research 9:304. doi: 10.12688/f1000research.23297.2, PMID: 32489650 PMC7222033

[ref55] Piatigorsky J. Wistow G. J. (1989). Enzyme/crystallins: gene sharing as an evolutionary strategy. Cell 57, 197–199. doi: 10.1016/0092-8674(89)90956-2, PMID: 2649248

[ref56] Piovani L. Leite D. J. Guerra L. A. Y. Simpson F. Musser J. M. Salvador-Martínez I.. (2023). Single-cell atlases of two lophotrochozoan larvae highlight their complex evolutionary histories. Sci. Adv. 9, eadg6034–eadg6016. doi: 10.1126/sciadv.adg6034, PMID: 37531419 PMC10396302

[ref57] Porath-Krause A. J. Pairett A. N. Faggionato D. Birla B. S. Sankar K. Serb J. M. (2016). Structural differences and differential expression among rhabdomeric opsins reveal functional change after gene duplication in the bay scallop, *Argopecten irradians* (Pectinidae). BMC Evol. Biol. 16:250. doi: 10.1186/s12862-016-0823-9, PMID: 27855630 PMC5114761

[ref58] Porter M. L. Blasic J. R. Bok M. J. Cameron E. G. Pringle T. Cronin T. W.. (2012). Shedding new light on opsin evolution. Proc. Biol. Sci. 279, 3–14. doi: 10.1098/rspb.2011.1819, PMID: 22012981 PMC3223661

[ref59] Ramirez M. D. Pairett A. N. Pankey M. S. Serb J. M. Speiser D. I. Swafford A. J.. (2016). The last common ancestor of bilaterian animals possessed at least 7 opsins. Genome Biol. Evol. 8, 3640–3652. doi: 10.1101/052902, PMID: 28172965 PMC5521729

[ref60] Randel N. Bezares-Calderón L. A. Gühmann M. Shahidi R. Jékely G. (2013). Expression dynamics and protein localization of rhabdomeric opsins in platynereis larvae. Integr. Comp. Biol. 53, 7–16. doi: 10.1093/icb/ict046, PMID: 23667045 PMC3687135

[ref61] Rawlinson K. A. Lapraz F. Ballister E. R. Terasaki M. Rodgers J. McDowell R. J.. (2019). Extraocular, rod-like photoreceptors in a flatworm express xenopsin photopigment. eLife 8, 1–28. doi: 10.7554/eLife.45465, PMID: 31635694 PMC6805122

[ref62] Rittschof D. Foward R. B. Jr. Cannon G. Welch J. M. McClary M. Jr. Holm E. R.. (1998). Cues and context: larval responses to physical and chemical cues. Biofouling 12, 31–44. doi: 10.1080/08927019809378344

[ref63] Rodriguez S. R. Ojeda F. P. Inestrosa N. C. (1993). Settlement of benthic marine invertebrates. Mar. Ecol. Prog. Ser. 97, 193–207. doi: 10.3354/meps097193

[ref64] Sadier A. Davies K. T. J. Yohe L. R. Yun K. Donat P. Hedrick B. P.. (2018). Multifactorial processes underlie parallel opsin loss in neotropical bats. eLife 7, 1–32. doi: 10.7554/eLife.37412, PMID: 30560780 PMC6333445

[ref65] Say T. E. Degnan S. M. (2020). Molecular and behavioural evidence that interdependent photo - and chemosensory systems regulate larval settlement in a marine sponge. Mol. Ecol. 29, 247–261. doi: 10.1111/mec.15318, PMID: 31791111

[ref66] Schneider T. Leitz T. (1994). Protein kinase C in hydrozoans: involvement in metamorphosis of Hydractinia and in pattern formation of Hydra. Roux’s Arch. Dev. Biol. Off. organ EDBO 203, 422–428. doi: 10.1007/BF00188691 28305948

[ref67] Senthilan P. R. Piepenbrock D. Ovezmyradov G. Nadrowski B. Bechstedt S. Pauls S.. (2012). Drosophila auditory organ genes and genetic hearing defects. Cell 150, 1042–1054. doi: 10.1016/j.cell.2012.06.043, PMID: 22939627

[ref68] Shen W. L. Kwon Y. Adegbola A. A. Luo J. Chess A. Montell C. (2011). Function of rhodopsin in temperature discrimination in Drosophila. Science 331, 1333–1336. doi: 10.1126/science.1198904, PMID: 21393546

[ref69] Shichida Y. Matsuyama T. (2009). Evolution of opsins and phototransduction. Philos. Trans. R. Soc. B-Biological Sci. 364, 2881–2895. doi: 10.1098/rstb.2009.0051, PMID: 19720651 PMC2781858

[ref70] Silver N. Cotroneo E. Proctor G. Osailan S. Paterson K. L. Carpenter G. H. (2008). Selection of housekeeping genes for gene expression studies in the adult rat submandibular gland under normal, inflamed, atrophic and regenerative states. BMC Mol. Biol. 9, 64–15. doi: 10.1186/1471-2199-9-64, PMID: 18637167 PMC2492873

[ref71] Smedley G. D. McElroy K. E. Serb J. M. (2022). Additive and epistatic effects influence spectral tuning in molluscan retinochrome opsin. J. Exp. Biol. 225:p.jeb242929. doi: 10.1101/2021.05.26.445805, PMID: 35531988

[ref72] Speiser D. I. Pankey M. Zaharoff A. K. Battelle B. A. Bracken-Grissom H. D. Breinholt J. W.. (2014). Using phylogenetically-informed annotation (PIA) to search for light-interacting genes in transcriptomes from non-model organisms. BMC Bioinfo. 15:350. doi: 10.1186/s12859-014-0350-x, PMID: 25407802 PMC4255452

[ref73] Sriram K. Insel P. A. (2018). G protein-coupled receptors as targets for approved drugs: how many targets and how many drugs? Mol. Pharmacol. 93, 251–258. doi: 10.1124/mol.117.111062, PMID: 29298813 PMC5820538

[ref74] Strader M. E. Aglyamova G. V. Matz M. V. (2018). Molecular characterization of larval development from fertilization to metamorphosis in a reef-building coral. BMC Genomics 19:17. doi: 10.1186/s12864-017-4392-0, PMID: 29301490 PMC5755313

[ref75] Takeuchi T. Kawashima T. Koyanagi R. Gyoja F. Tanaka M. Ikuta T.. (2012). Draft genome of the pearl oyster Pinctada fucata: a platform for understanding bivalve biology. DNA Res. 19, 117–130. doi: 10.1093/dnares/dss005, PMID: 22315334 PMC3325083

[ref76] Terakita A. (2005). The opsins. Genome Biol. 6:213. doi: 10.1186/gb-2005-6-3-213, PMID: 15774036 PMC1088937

[ref77] Terakita A. Hara R. Hara T. (1989). Retinal-binding protein as a shuttle for retinal in the rhodopsin-retinochrome system of the squid visual cells. Vis. Res. 29, 639–652. doi: 10.1016/0042-6989(89)90026-6, PMID: 2626821

[ref78] Terakita A. Nagata T. (2014). Functional properties of opsins and their contribution to light-sensing physiology. Zool. Sci. 31, 653–659. doi: 10.2108/zs140094, PMID: 25284384

[ref79] Tran C. Hadfield M. G. (2012). Are G-protein-coupled receptors involved in mediating larval settlement and metamorphosis of coral planulae? Biol. Bull. 222, 128–136. doi: 10.1086/BBLv222n2p128, PMID: 22589403

[ref1002] Trapido-Rosenthal H. G. Morse D. E. (1986). Availability of chemosensory receptors is down-regulated by habituation of larvae to a morphogenetic signal. Proc Natl Acad Sci. USA 83, 7658–7662. doi: 10.1073/pnas.83.20.7658, PMID: 3020553 PMC386780

[ref80] True J. R. Carroll S. B. (2002). Gene co-option in physiological and morphological evolution. Annu. Rev. Cell Dev. Biol. 18, 53–80. doi: 10.1146/annurev.cellbio.18.020402.140619 12142278

[ref81] Velloso J. P. L. Ascher D. B. Pires D. E. V. (2021). pdCSM-GPCR: predicting potent GPCR ligands with graph-based signatures. Bioinforma. Adv. 1, 1–7. doi: 10.1093/bioadv/vbab031, PMID: 34901870 PMC8651072

[ref82] Venkatakrishnan A. J. Deupi X. Lebon G. Tate C. G. Schertler G. F. Babu M. M. (2013). Molecular signatures of G-protein-coupled receptors. Nature 494, 185–194. doi: 10.1038/nature11896 23407534

[ref83] Vizueta J. Sánchez-Gracia A. Rozas J. (2020). Bitacora: a comprehensive tool for the identification and annotation of gene families in genome assemblies. Mol. Ecol. Resour. 20, 1445–1452. doi: 10.1111/1755-0998.13202, PMID: 32492257

[ref84] Vöcking O. Kourtesis I. Hausen H. (2015). Posterior eyespots in larval chitons have a molecular identity similar to anterior cerebral eyes in other bilaterians. EvoDevo 6, 40–14. doi: 10.1186/s13227-015-0036-0, PMID: 26702352 PMC4689004

[ref85] Vöcking O. Kourtesis I. Tumu S. C. Hausen H. (2017). Co-expression of xenopsin and rhabdomeric opsin in photoreceptors bearing microvilli and cilia. eLife 6, 1–26. doi: 10.7554/eLife.23435, PMID: 28876222 PMC5648526

[ref86] Vöcking O. Leclère L. Hausen H. (2021). The rhodopsin-retinochrome system for retinal re-isomerization predates the origin of cephalopod eyes. BMC Ecol. Evol. 21, 215–214. doi: 10.1186/s12862-021-01939-x, PMID: 34844573 PMC8628405

[ref87] Vöcking O. Macias-Muñoz A. Jaeger S. J. Oakley T. H. (2022). Deep diversity: extensive variation in the components of complex visual systems across animals. Cells 11, 1–23. doi: 10.3390/cells11243966, PMID: 36552730 PMC9776813

[ref88] Waller T. R. (1981). Functional morphology and development of veliger larvae of the European oyster, *Ostrea edulis* Linne. Smithson. Contrib. to Zool. 1–70, 1–70. doi: 10.5479/si.00810282.328

[ref89] Wollesen T. McDougall C. Arendt D. (2019). Remnants of ancestral larval eyes in an eyeless mollusk? Molecular characterization of photoreceptors in the scaphopod Antalis entalis. EvoDevo 10, 25–12. doi: 10.1186/s13227-019-0140-7, PMID: 31641428 PMC6800502

[ref90] Yurchenko O. V. Skiteva O. I. Voronezhskaya E. E. Dyachuk V. A. (2018). Nervous system development in the Pacific oyster, *Crassostrea gigas* (Mollusca: Bivalvia). Front. Zool. 15, 10–21. doi: 10.1186/s12983-018-0259-8, PMID: 29681988 PMC5896133

[ref91] Zeng Z. Jiang C. Tan Q. Tang B. Huang Z. (2022). Larvae of a marine gastropod and a marine bivalve share common gene expression signatures during metamorphic competence. Mar. Biol. 169, 1–11. doi: 10.1007/s00227-022-04106-y

