# Supplementary material for: Opsin expression varies across larval development and taxa in pteriomorphian bivalves
Source: Front Neurosci. 2024 Mar 18;18:1357873. doi: 10.3389/fnins.2024.1357873 (PMC10982516; doi:10.3389/fnins.2024.1357873)
Supplement: Supplementary file 1 [file Data_Sheet_1.PDF]

```
#NEXUS
begin trees;
	tree tree_1 = [&R] ((Cale-melatonin_1BB:0.366916,((Tgra-
melatonin_1BB:0.070025,(Sbro-melatonin_1BB:0.023063,Skag-
melatonin_1BB:0.020766)[&ufboot=100]:0.100474)[&ufboot=100]:0.803328,
(((Ajap_mel:0.241951,(Pmag_mel:0.134749,
('Cfar.melatonin.PE':0.089678,Pyes-melatonin_1BB:0.054912)
[&ufboot=100]:0.053819)[&ufboot=100]:0.049299)[&ufboot=98]:0.038797,
((Apur_mel:1.0E-6,Airr-melatonin_1BB:1.0E-6)[&ufboot=100]:0.314783,
(Aple_mel:0.050392,
('Pmax.melatonin.PE':2.0E-6,'Pmax.melatonin.1BB.PE':3.0E-6)
[&ufboot=100]:0.060086)[&ufboot=100]:0.056483)[&ufboot=95]:0.038988)
[&ufboot=100]:0.672428,((Pvir-melatonin_1BB:0.609448,
((Mgal_mel:3.0E-6,Mgal-melatonin_1BB:1.0E-6)[&ufboot=100]:0.077739,
(Mcal_mel:0.021196,(Mcor_mel:1.0E-6,Mcor-melatonin_1BB:1.0E-6)
[&ufboot=100]:0.029094)[&ufboot=100]:0.063994)[&ufboot=100]:0.345663)
[&ufboot=100]:0.260402,('Pfuc.melatonin.MA':0.514339,(Oedu-
melatonin_1BB:0.137328,(Cvir-melatonin_1BB:0.200183,(Cari-
melatonin_1BB:0.054678,('Cang.melatonin.OS':0.005116,
('Cgig.melatonin.OS':1.0E-6,'Cgig.melatonin.1BB.OS':1.0E-6)
[&ufboot=100]:0.009816)[&ufboot=98]:0.049912)[&ufboot=98]:0.095476)
[&ufboot=63]:0.10215)[&ufboot=100]:0.491768)[&ufboot=100]:0.136051)
[&ufboot=100]:0.354016)[&ufboot=96]:0.191582)[&ufboot=92]:0.180489)
[&ufboot=100]:1.627729,(((('Cvir-pseudopsin|
XP_022337418.1':0.121185,'Oedu-pseudopsin|XP_048752667.1':0.115273)
[&ufboot=90]:0.033541,(Cari-pseudopsin:0.038704,
('Cang.pseudopsin.OS':1.0E-6,'Cgig.pseudopsin.OS':1.0E-6)
[&ufboot=100]:0.026822)[&ufboot=100]:0.049575)[&ufboot=100]:0.424043,
(('Pfuc.pseudopsin.MA':0.903776,(Pvir-pseudopsin:0.34747,
((Mgal_pseudopn:0.008706,'Medu.pseudopsin|CAG2213510.1.MY':0.011262)
[&ufboot=100]:0.060666,(Mcal_pseudopn:0.010114,
(Mcor_pseudopn:1.0E-6,'Mcor-pseudopsin|CAC5360081.1':1.0E-6)
[&ufboot=100]:0.007139)[&ufboot=100]:0.0327)[&ufboot=100]:0.337201)
[&ufboot=100]:0.587162)[&ufboot=94]:0.123725,((Tgra-
pseudopsin:0.050573,(Sbor-pseudopsin:2.0E-6,Skag-pseudopsin:0.005846)
[&ufboot=100]:0.049467)[&ufboot=100]:0.414685,(Cale-
pseudopsin:0.353945,(Ajap_pseudopn:0.045144,
(((Aple_pseudopn:0.027961,'Pmax.pseudopsin|
XP_033749045.1.PE':0.032677)[&ufboot=100]:0.010116,
(Apur_pseudopn:0.002696,Airr-pseudopsin:1.0E-6)[&ufboot=100]:0.112591)
[&ufboot=100]:0.035673,(Pmag_pseudopn:0.072301,
(Mvar_pseudopn:0.045437,('Cfar.pseudopsin.PE':0.048123,'Pyes-
pseudopsin|XP_021367303.1':0.004931)[&ufboot=100]:0.006322)
[&ufboot=100]:0.0375)[&ufboot=99]:0.020306)[&ufboot=100]:0.021507)
[&ufboot=100]:0.210357)[&ufboot=100]:0.184449)[&ufboot=79]:0.106557)
[&ufboot=75]:0.122468)[&ufboot=100]:2.044704,
(('Placopsin_XP_002113363.1':2.576101,'Placopsin_XP_002112437.1':2.226
53)[&ufboot=100]:0.7754,((((Cale-opnGq-nc1:0.861108,((Azeb-opnGq-
nc3:0.316886,(Sbro-opnGq-nc3:1.0E-6,Skag-opnGq-nc3:0.015771)
[&ufboot=100]:0.427502)[&ufboot=100]:0.324313,(Oedu-opnGq-
```

nc1:0.228582,(Cvir-opnGq-nc1:0.118241,(Cari-opnGq-nc1:3.0E-6,
('Cgig.opnGq-nc.A.OS':0.015238,'Cang.opnGq-nc.A.OS':0.018827)
[&ufboot=100]:0.040092)[&ufboot=100]:0.084354)[&ufboot=100]:0.235071)
[&ufboot=100]:0.927692)[&ufboot=99]:0.311037)[&ufboot=100]:1.226154,
((Pvir-opnGq-nc:0.717941,((Mcal-000024:0.032157,'Myco.opnGq-
nc.B.MY':0.036458)[&ufboot=100]:0.05011,('Medu.opnGq-
nc.B.MY':0.030891,Mgal-opnGq-nc:0.007088)[&ufboot=100]:0.104781)
[&ufboot=100]:0.43229)[&ufboot=100]:1.045903,(('Pfuc.opnGq-
nc.B.MA':1.054549,(Oedu-opnGq-nc2:0.201677,(Cvir-opnGq-nc2:0.153309,
(Cari-opnGq-nc2:0.060913,('Cgig.opnGq-nc.B.OS':1.0E-6,'Cang.opnGq-
nc.B.OS':0.008718)[&ufboot=100]:0.036839)[&ufboot=100]:0.10924)
[&ufboot=100]:0.278296)[&ufboot=100]:0.947392)[&ufboot=99]:0.215659,
(((Sbro-opnGq_nc2:0.037433,Skag-opnGq-nc2:0.011231)
[&ufboot=100]:1.056117,((Tgra-opnGq-nc4:0.341295,(Sbro-opnGq-
nc4:0.164978,Skag-opnGq-nc4:0.076835)[&ufboot=100]:0.252798)
[&ufboot=100]:0.668178,((Sbro-opnGq_nc1:0.182397,Tgra-opnGq-
nc1:0.281981)[&ufboot=100]:0.553337,(Azeb-opnGqn-c1:0.270022,Azeb-
opnGq-nc2:0.347496)[&ufboot=100]:0.496991)[&ufboot=95]:0.159023)
[&ufboot=100]:0.219982)[&ufboot=100]:0.294357,(Cale-opnGq-
nc2:0.925233,((('Pmax.opnGq-nc.B.PE.2':0.233483,(Aple-opnGq-
nc2:0.125365,(Airr-opnGq-nc2:1.0E-6,Apur-opnGqn-c2:1.0E-6)
[&ufboot=100]:0.292906)[&ufboot=100]:0.048482)[&ufboot=100]:0.434977,
(Pmag-opnGq-nc2:0.296438,(Pyes-opnGq-nc2:0.195801,('Cfar.opnGq-
nc.B.PE.2':0.226437,Mvar-opnGq-nc2:0.266464)[&ufboot=98]:0.045327)
[&ufboot=99]:0.185377)[&ufboot=98]:0.091865)[&ufboot=100]:1.100936,
(Pmag-opnGq-nc1:0.262916,((Pyes-opnGq-nc1:0.124731,('Cfar.opnGq-
nc.B.PE.1':0.153626,Mvar-opnGq-nc1:0.115923)[&ufboot=99]:0.039573)
[&ufboot=100]:0.124147,(Ajap-opnGq-nc1:0.109621,((Airr-opnGq-
nc1:0.010439,Apur-opnGq-nc1:0.044036)[&ufboot=100]:0.114828,
('Pmax.opnGq-nc.B.PE.1':0.086041,Aple-opnGq-nc1:0.037502)
[&ufboot=100]:0.032718)[&ufboot=100]:0.123728)[&ufboot=99]:0.056053)
[&ufboot=53]:0.05828)[&ufboot=100]:0.534466)[&ufboot=98]:0.160163)
[&ufboot=100]:0.592652)[&ufboot=98]:0.196141)[&ufboot=99]:0.266807)
[&ufboot=100]:0.399883)[&ufboot=100]:0.458561,((Pmag-opnGq1:0.104819,
((Mvar-opnGq1:0.075384,('Cfar.opnGq.A.PE.1':0.048505,Pyes-
opnGq1:0.082676)[&ufboot=100]:0.040925)[&ufboot=100]:0.088948,(Ajap-
opnGq1:0.226482,((Airr-opnGq1:0.031528,Apur-opnGq1:0.037137)
[&ufboot=100]:0.173357,('Pmax.opnGq.A.PE.1':0.061361,Aple-
opnGq1:0.064118)[&ufboot=100]:0.032349)[&ufboot=100]:0.098441)
[&ufboot=100]:0.052317)[&ufboot=100]:0.070097)[&ufboot=100]:0.615258,
((('Pfuc.opnGq.B.MA':0.353831,(Oedu-opnGq-unk:0.257729,(Oedu-
opnGq:0.156932,(Cvir-opnGq:0.048005,(Cari-opnGq:0.021021,
('Cgig.opnGq.B.OS':0.002956,'Cang.opnGq.B.OS':0.004177)
[&ufboot=100]:0.022532)[&ufboot=100]:0.115871)[&ufboot=100]:0.129517)
[&ufboot=95]:0.058097)[&ufboot=100]:0.319624)[&ufboot=86]:0.092636,
((Pvir-opnGq3:0.267113,
((Mcal-000023:0.010734,'Myco.opnGq.B.MY.1':0.00682)
[&ufboot=100]:0.014248,('Medu.opnGq.B.MY.1':0.017717,Mgal-
opnGq3:0.008638)[&ufboot=100]:0.02176)[&ufboot=100]:0.195536)
[&ufboot=100]:0.384315,((Pvir-opnGq1:0.166413,

((Mcal-000021:0.020314,'Myco.opnGq.B.MY.2':0.022683)
[&ufboot=100]:0.029304,('Medu.opnGq.B.MY.2':0.034597,Mgal-
opnGq1:0.030491)[&ufboot=100]:0.051563)[&ufboot=100]:0.212347)
[&ufboot=100]:0.400666,(Pvir-opnGq2:0.364341,
((Mcal-000022:0.018498,'Myco.opnGq.B.MY.3':0.017131)
[&ufboot=100]:0.037389,('Medu.opnGq.B.MY.3':0.048522,Mgal-
opnGq2:0.018172)[&ufboot=94]:0.021081)[&ufboot=100]:0.271435)
[&ufboot=100]:0.150741)[&ufboot=98]:0.108006)[&ufboot=100]:0.185727)
[&ufboot=49]:0.060302,(Cale-opnGq:0.512274,((Azeb-opnGq:0.140384,
(Tgra-opnGq:0.098227,(Sbro-opnGq:3.0E-6,Skag-opnGq:0.028874)
[&ufboot=100]:0.076684)[&ufboot=100]:0.123283)[&ufboot=100]:0.48772,
((Pmag-opnGq4:0.091557,((Mvar-opnGq4:0.061122,
('Cfar.opnGq.B.PE.1':0.04723,Pyes-opnGq4:0.039521)
[&ufboot=100]:0.02941)[&ufboot=100]:0.046133,(Ajap-opnGq4:0.129,
((Airr-opnGq4:0.028098,Apur-opnGq4:0.045426)[&ufboot=100]:0.1115,
('Pmax.opnGq.B.PE.1':0.038511,Aple-opnGq4:0.053408)
[&ufboot=100]:0.005991)[&ufboot=100]:0.041202)[&ufboot=100]:0.033903)
[&ufboot=60]:0.015146)[&ufboot=100]:0.344648,((Mvar-opnGq2:0.007349,
(('Cfar.opnGq.B.PE.2':0.014643,Pyes-opnGq2:0.065976)
[&ufboot=100]:0.032331,(Pmag-opnGq2:0.078742,(Ajap-opnGq2:0.040272,
((Airr-opnGq2:0.025621,Apur-opnGq2:0.011254)[&ufboot=100]:0.035257,
('Pmax.opnGq.B.PE.2':0.134654,Aple-opnGq2:0.019886)
[&ufboot=100]:0.017647)[&ufboot=86]:0.015471)[&ufboot=100]:0.065099)
[&ufboot=100]:0.028261)[&ufboot=100]:0.022039)[&ufboot=100]:0.2496,
(((Airr-opnGq3:0.032972,Apur-opnGq3:0.017502)[&ufboot=100]:0.105726,
('Pmax.opnGq.B.PE.3':0.057471,Aple-opnGq3:0.04848)
[&ufboot=97]:0.014245)[&ufboot=97]:0.025009,(Ajap-opnGq3:0.089676,
(Pmag-opnGq3:0.09296,(Pyes-opnGq3:0.039365,
('Cfar.opnGq.B.PE.3':0.027615,Mvar-opnGq3:0.033118)
[&ufboot=96]:0.013037)[&ufboot=100]:0.072293)[&ufboot=87]:0.015984)
[&ufboot=87]:0.013737)[&ufboot=100]:0.311042)[&ufboot=100]:0.199088)
[&ufboot=100]:0.240168)[&ufboot=48]:0.087292)[&ufboot=50]:0.086471)
[&ufboot=84]:0.175762)[&ufboot=100]:0.739677)[&ufboot=100]:0.523241,
(((((Azeb-peropsin1:0.505338,(Tgra-peropsin:0.240746,(Skag-peropsin-
a:0.010209,(Sbro-peropsin:0.006426,Skag-peropsin-b:0.026434)
[&ufboot=80]:0.009618)[&ufboot=100]:0.113371)[&ufboot=100]:0.479772)
[&ufboot=100]:0.567342,('Pfuc.peropsin.MA':0.565393,(Oedu-
peropsin:0.24437,(Civr-peropsin:0.215181,('Cang.peropsin.OS':2.0E-6,
('Cgig.peropsin.OS':0.007425,Cari-peropsin:0.075897)
[&ufboot=97]:0.014545)[&ufboot=97]:0.087784)[&ufboot=100]:0.148486)
[&ufboot=100]:0.961955)[&ufboot=100]:0.606833)[&ufboot=100]:0.920549,
(((Pvir-RTC:0.29039,((Mcal-000006:0.013646,'Myco.RTC.MY':0.022249)
[&ufboot=54]:1.0E-6,('Medu.RTC.MY':0.006316,Mgal-RTC:0.007845)
[&ufboot=100]:0.089931)[&ufboot=98]:0.449716)[&ufboot=100]:1.219562,
('Pfuc.RTC.MA':0.845257,(Oedu-RTC:0.313585,(Cvir-RTC:0.242354,(Cari-
RTC:0.113321,('Cgig.RTC.OS':1.0E-6,'Cang.RTC.OS':0.007235)
[&ufboot=100]:0.129226)[&ufboot=100]:0.151122)[&ufboot=100]:0.209778)
[&ufboot=100]:0.64035)[&ufboot=97]:0.286895)[&ufboot=99]:0.214651,
((Azeb-RTC:0.185024,(Sbro-RTC:0.047234,Tgra-RTC:0.169677)
[&ufboot=98]:0.158533)[&ufboot=100]:0.727777,(Cale-RTC:0.735184,(Pyes-

RTC:0.010577,('Cfar.RTC.PE':0.026678,(Mvar-RTC:0.071136,(Pmag-
RTC:0.087981,(Ajap-RTC:0.196534,((Airr-RTC:0.039147,Apur-RTC:0.037039)
[&ufboot=100]:0.076659,('Pmax.RTC.PE':0.023127,Aple-RTC:0.003698)
[&ufboot=100]:0.035085)[&ufboot=100]:0.068159)[&ufboot=100]:0.03307)
[&ufboot=100]:0.069749)[&ufboot=94]:0.025401)[&ufboot=94]:0.029369)
[&ufboot=100]:1.147251)[&ufboot=99]:0.333876)[&ufboot=55]:0.028771)
[&ufboot=100]:2.983278)[&ufboot=100]:0.740912,((((Pvir-opn5a:1.21116,
('Myco.opn5.MY.1':0.087316,(Mcal-000001:0.064405,
('Medu.opn5.MY.1':0.037198,Mgal-opn5a:0.014645)[&ufboot=100]:0.101289)
[&ufboot=76]:0.052463)[&ufboot=98]:0.509203)[&ufboot=100]:0.281934,
(Pvir-opn5b:0.433309,((Mcal-000003:0.004725,
('Myco.opn5.MY.2':0.021962,('Medu.opn5.MY.2':0.041664,Mgal-
opn5c:0.060195)[&ufboot=100]:0.055619)[&ufboot=98]:0.006915)
[&ufboot=99]:0.251544,
(((Mcal-000002:0.041863,'Myco.opn5.MY.3':0.088678)
[&ufboot=100]:0.084983,('Medu.opn5.MY.3':0.096692,Mgal-opn5b:0.043196)
[&ufboot=100]:0.132992)[&ufboot=100]:0.57988,
((Mcal-000005:0.033726,'Myco.opn5.MY.4':0.053541)
[&ufboot=100]:0.034591,(Mcal-000004:0.09837,
('Medu.opn5.MY.4':0.016226,Mgal-opn5d:0.014957)[&ufboot=100]:0.047257)
[&ufboot=100]:0.045363)[&ufboot=100]:0.24591)[&ufboot=97]:0.118221)
[&ufboot=98]:0.22482)[&ufboot=100]:0.208729)[&ufboot=100]:0.724622,
((Cale-opn5:0.854676,(Pyes-opn5:0.052722,('Cfar.opn5.PE':0.023636,
(Mvar-opn5:0.069448,(Pmag-opn5:0.130955,(Ajap-opn5:0.076833,((Airr-
opn5:0.027481,Apur-opn5:0.016111)[&ufboot=100]:0.168647,
('Pmax.opn5.PE':0.103174,Aple-opn5:0.066294)[&ufboot=99]:0.052825)
[&ufboot=100]:0.188765)[&ufboot=95]:0.061809)[&ufboot=91]:0.055452)
[&ufboot=46]:0.01192)[&ufboot=48]:0.022947)[&ufboot=100]:0.735626)
[&ufboot=95]:0.262225,(('Pfuc.opn5.MA':0.599262,(Cvir-opn5:0.114774,
(Oedu-opn5:0.306976,(Cari-opn5:0.041199,
('Cgig.opn5.OS':0.014752,'Cang.opn5.OS':1.0E-6)[&ufboot=100]:0.019606)
[&ufboot=100]:0.211395)[&ufboot=87]:0.094164)[&ufboot=100]:0.70128)
[&ufboot=100]:0.46127,((Tgra-opn5b:0.126626,(Sbro-opn5b:0.049981,Skag-
opn5b:0.039672)[&ufboot=100]:0.09278)[&ufboot=100]:0.403476,(Azeb-
opn5a:0.200741,(Tgra-opn5a:0.114932,(Sbro-opn5a:0.080278,Skag-
opn5a:0.007296)[&ufboot=100]:0.063221)[&ufboot=100]:0.251071)
[&ufboot=100]:0.113438)[&ufboot=100]:0.521289)[&ufboot=98]:0.221016)
[&ufboot=99]:0.336522)[&ufboot=100]:1.731548,
((('Pfuc.opnGo.A.MA.1':1.957126,(Azeb-opnGo3:1.099219,
('Tgra_opsin111.t1':0.176121,
('Sbro_opsin176.t1':0.039532,'Skag_opsin105.t1':0.006288)
[&ufboot=100]:0.132726)[&ufboot=100]:0.449136)[&ufboot=100]:1.145901)
[&ufboot=100]:1.414686,((Cales-opnGo:2.713956,
(('Pfuc.opnGo.A.MA.2b':0.852722,'Pfuc.opnGo.A.MA.2a':1.075667)
[&ufboot=96]:0.345349,(Oedu-opnGo:0.591394,(Cvir-opnGo:0.549918,(Cari-
opnGo:0.135661,('Cgig.opnGo.A.OS':0.005325,'Cang.opnGo.A.OS':0.003375)
[&ufboot=100]:0.050354)[&ufboot=100]:0.435814)[&ufboot=89]:0.25317)
[&ufboot=100]:0.962184)[&ufboot=78]:0.294416)[&ufboot=97]:0.434889,
((Airr-opnGo1:0.072045,Apur-opnGo1:0.016753)[&ufboot=100]:0.106932,
(('Pmax.opnGo.A.PE':0.110529,Aple-opnGo1:0.165403)

[&ufboot=100]:0.088004,(Ajap-opnGo1:0.253666,(Pmag-opnGo1:0.426268,
(Mvar-opnGo1:0.353222,('Cfar.opnGo.A.PE':0.155295,Pyes-
opnGo1:0.174235)[&ufboot=100]:0.148788)[&ufboot=99]:0.14974)
[&ufboot=94]:0.032753)[&ufboot=94]:0.239046)[&ufboot=94]:0.153881)
[&ufboot=100]:1.38297)[&ufboot=100]:0.355596)[&ufboot=100]:0.676784,
(('Pfuc.opnGo.B.MA':1.016642,(((Airr-opnGo2:0.056937,Apur-
opnGo2:0.094934)[&ufboot=100]:0.319638,
('Pmax.opnGo.B.PE':0.189981,Aple-opnGo2:0.140368)
[&ufboot=100]:0.179772)[&ufboot=99]:0.099091,(Ajap-opnGo2:0.320878,
(Pmag-opnGo2:0.341944,(Mvar-opnGo2:0.232492,
('Cfar.opnGo.B.PE':0.151847,Pyes-opnGo2:0.087337)
[&ufboot=100]:0.039224)[&ufboot=100]:0.064121)[&ufboot=100]:0.175717)
[&ufboot=74]:0.058772)[&ufboot=100]:0.655238)[&ufboot=97]:0.124436,
((Azeb-opnGo2:0.837049,(Azeb-opnGo1:0.35752,(Tgra-opnGo:0.174903,
(Sbro-opnGo:0.067055,Skag-opnGo1:0.031837)[&ufboot=100]:0.212346)
[&ufboot=100]:0.54518)[&ufboot=100]:0.525051)[&ufboot=100]:0.334171,
((((Mcal-000007:0.026028,'Myco.opnGo.B.MY.1':0.042948)
[&ufboot=98]:0.024061,('Medu.opnGo.B.MY.1':0.051666,Mgal-
opnGo1:0.026988)[&ufboot=100]:0.143676)[&ufboot=100]:0.373564,
(Mcal-000008:0.054492,('Myco.opnGo.B.MY.2':0.031878,
('Medu.opnGo.B.MY.2':0.044681,Mgal-opnGo2:0.041307)
[&ufboot=100]:0.137706)[&ufboot=98]:0.019617)[&ufboot=100]:0.250354)
[&ufboot=100]:0.732725,(Pvir-opnGo1:1.173898,(Pvir-opnGo2:0.539365,
(('Myco.opnGo.B.MY.3':0.006939,('Mcal_opsin-035.t1|opnGo3':0.025506,
('Medu.opnGo.B.MY.3':0.043548,Mgal-opnGo3:0.017685)
[&ufboot=100]:0.104281)[&ufboot=100]:0.015647)[&ufboot=100]:0.447996,
(Pvir-opnGo3:0.813141,((Mcal-000010:1.0E-6,Mcal-000011:1.0E-6)
[&ufboot=100]:0.009786,('Myco.opnGo.B.MY.4':0.027241,(Mgal-
opnGo4:1.0E-6,('Medu.opnGo.B.MY.4':0.013441,Mgal-opnGo5:0.00672)
[&ufboot=100]:0.027093)[&ufboot=100]:0.092701)[&ufboot=94]:0.011383)
[&ufboot=100]:0.537978)[&ufboot=100]:0.356619)[&ufboot=98]:0.117179)
[&ufboot=100]:0.517012)[&ufboot=100]:0.186285)[&ufboot=100]:0.492805)
[&ufboot=98]:0.091641)[&ufboot=100]:1.364574)[&ufboot=91]:0.196705)
[&ufboot=96]:0.080644)[&ufboot=99]:0.229451,
(('Pfuc.opnGx.A.MA':2.084343,((Cale-opnGx1:1.086227,(Tgra-
opnGx2:0.295866,(Sbro-opnGx2:0.011066,Skag-opnGx2:0.011084)
[&ufboot=100]:0.177375)[&ufboot=100]:1.190631)[&ufboot=72]:0.171854,
((Ajap-opnGx1b:0.405106,((Mvar-opnGx1b:0.15682,
('Cfar.opnGx.A.PE.1':0.26344,Pyes-opnGx1b:0.175057)
[&ufboot=100]:0.075419)[&ufboot=100]:0.282085,((Airr-
opnGx1b:0.077331,Apur-opnGx1b:0.147537)[&ufboot=100]:0.33256,
('Pmax.opnGx.A.PE.1':0.129712,Aple-opnGx1b:0.12307)
[&ufboot=100]:0.185776)[&ufboot=84]:0.17874)[&ufboot=82]:0.131166)
[&ufboot=100]:1.135757,((((Pvir-opnGx1:0.290879,(Pvir-
opnGx2:0.756872,Pvir-opnGx3:0.781253)[&ufboot=100]:0.369481)
[&ufboot=99]:0.065318,
((Mcal-000012:0.011947,'Myco.opnGx.A.MY.1':0.038389)
[&ufboot=100]:0.031672,('Medu.opnGx.A.MY.1':0.025693,Mgal-
opnGx1:0.015975)[&ufboot=100]:0.03886)[&ufboot=100]:0.314911)
[&ufboot=100]:0.41585,((Pvir-opnGx4:1.434817,(Pvir-

opnGx5:0.025808,Pvir-opnGx6:0.001002)[&ufboot=100]:0.947552)
[&ufboot=100]:0.429254,
(('Medu.opnGx.A.MY.2b':0.170754,'Myco.opnGx.A.MY.2':0.158664)
[&ufboot=100]:0.77039,(Mcal-000013:0.054408,
('Medu.opnGx.A.MY.2a':0.01703,Mgal-opnGx2:0.014518)
[&ufboot=100]:0.108087)[&ufboot=100]:0.436658)[&ufboot=99]:0.201182)
[&ufboot=100]:1.160648)[&ufboot=100]:0.366101,
(('Pfuc.opnGx.A1.MA.1a':0.908368,'Pfuc.opnGx.A1.MA.1b':0.479558)
[&ufboot=100]:0.535363,(((Sbro-opnGx11:0.319231,Tgra-opnGx11:0.248443)
[&ufboot=100]:0.615865,(Azeb-opnGx9:0.726707,(Azeb-opnGx1:0.260692,
(Tgra-opnGx1:0.099267,(Sbro-opnGx1:0.027782,Skag-opnGx1:0.015174)
[&ufboot=100]:0.054869)[&ufboot=100]:0.364559)[&ufboot=100]:0.32161)
[&ufboot=100]:0.324553)[&ufboot=100]:0.27063,(((Pmag-opnGx1a:0.184008,
(Mvar-opnGx1a:0.057359,('Cfar.opnGx.A.PE.2':0.066546,Pyes-
opnGx1a:0.086057)[&ufboot=79]:0.028177)[&ufboot=100]:0.103338)
[&ufboot=99]:0.051614,(('Pmax.opnGx.A.PE.2':0.126751,Aple-
opnGx1a:0.087053)[&ufboot=100]:0.073023,(Ajap-opnGx1a:0.416416,(Airr-
opnGx1a:0.088488,Apur-opnGx1a:0.068538)[&ufboot=100]:0.215088)
[&ufboot=32]:0.086313)[&ufboot=32]:0.060951)[&ufboot=100]:0.988172,
((Oedu-opnGx2:0.248618,((Cvir-opnGx1a:3.0E-6,Cvir-opnGx1b:0.004596)
[&ufboot=100]:0.107388,(Cari-opnGx1:0.035065,
('Cgig.opnGx.A.OS.1':1.0E-6,'Cang.opnGx.A.OS.1':0.00899)
[&ufboot=100]:0.02434)[&ufboot=100]:0.046116)[&ufboot=100]:0.211539)
[&ufboot=100]:0.125484,(Oedu-opnGx1:0.403492,((Cvir-
opnGx2a:0.005103,Cvir-opnGx2b:0.0031)[&ufboot=100]:0.267627,(Cari-
opnGx2:0.060241,
('Cgig.opnGx.A.OS.2':0.016922,'Cang.opnGx.A.OS.2':0.008186)
[&ufboot=100]:0.072665)[&ufboot=100]:0.222518)[&ufboot=100]:0.25113)
[&ufboot=100]:0.48237)[&ufboot=100]:0.774985)[&ufboot=66]:0.132046)
[&ufboot=67]:0.20628)[&ufboot=75]:0.099625)[&ufboot=82]:0.176127)
[&ufboot=69]:0.075975)[&ufboot=100]:0.464961)[&ufboot=97]:0.290836,
(((Azeb-opnGx3:1.531092,(Skag-opnGx9b:0.056829,(Sbro-
opnGx9:0.006148,Skag-opnGx9a:0.022843)[&ufboot=98]:0.006181)
[&ufboot=100]:1.657964)[&ufboot=99]:0.378791,(Azeb-opnGx2:1.230328,
((('Myco.opnGx.B1.MY':0.280753,(Pvir-opnGx9:0.403373,
('Mcal_opsin-089.t1|Xenopsin':0.138696,
('Medu.opnGx.B1.MY':0.033663,Mgal-opnGx9:0.031482)
[&ufboot=100]:0.159349)[&ufboot=100]:0.152721)[&ufboot=100]:0.232547)
[&ufboot=100]:2.135204,('Pfuc.opnGx.B1.MA':1.578243,(Oedu-
opnGx3:0.288499,(Cvir-opnGx3:0.234658,(Cari-opnGx3:0.050113,
('Cgig.opnGx.B1.OS':0.019422,'Cang.opnGx.B1.OS':0.017954)
[&ufboot=100]:0.085378)[&ufboot=100]:0.296494)[&ufboot=100]:0.200469)
[&ufboot=100]:1.101652)[&ufboot=98]:0.230345)[&ufboot=99]:0.293104,
((Cale-opnGx3:0.765443,(Mvar-opnGx2:0.102615,
(('Cfar.opnGx.B1.PE.1':0.056454,Pyes-_opnGx2:0.102729)
[&ufboot=98]:0.020889,((Ajap-opnGx2:0.123053,Pmag-opnGx2:0.249694)
[&ufboot=96]:0.050425,((Air-opnGx2:0.041058,Apur-opnGx2:0.047861)
[&ufboot=100]:0.174345,('Pmax.opnGx.B1.PE.1':0.066648,Aple-
opnGx2:0.093598)[&ufboot=100]:0.055609)[&ufboot=100]:0.150718)
[&ufboot=92]:0.08189)[&ufboot=90]:0.023083)[&ufboot=100]:0.493796)

[&ufboot=100]:0.292159,(((Mvar-opnGx3b:0.208991,
('Cfar.opnGx.B1.PE.2':0.196182,Pyes-opnGx3b:0.168342)
[&ufboot=99]:0.070778)[&ufboot=100]:0.186332,((Pmag-
opnGx3c:0.005512,Pmag-opnGx3d:0.005062)[&ufboot=100]:0.283472,(Ajap-
opnGx3b:0.370676,((Airr-opnGx3b:0.054008,Apur-opnGx3b:0.110669)
[&ufboot=100]:0.326555,('Pmax.opnGx.B1.PE.2':0.148824,Aple-
opnGx3b:0.14555)[&ufboot=100]:0.142279)[&ufboot=100]:0.371039)
[&ufboot=83]:0.091009)[&ufboot=83]:0.077885)[&ufboot=100]:1.094,
((((Airr-opnGx3a:0.066245,Apur-opnGx3a:0.059188)
[&ufboot=100]:0.342066,('Pmax.opnGx.B1.PE.3':0.09505,Aple-
opnGx3a:0.077299)[&ufboot=100]:0.125787)[&ufboot=100]:0.266828,(Ajap-
opnGx3a:0.177751,(Pyes-opnGx3a:0.076488,
('Cfar.opnGx.B1.PE.3':0.125134,Mvar-opnGx3a:0.168111)
[&ufboot=99]:0.034496)[&ufboot=100]:0.258302)[&ufboot=66]:0.070061)
[&ufboot=100]:0.387071,((Ajap-opnGx3c:0.226267,((Airr-
opnGx3c:1.0E-6,Apur-opnGx3c:1.0E-6)[&ufboot=100]:0.258153,
('Pmax.opnGx.B1.PE.4':0.044659,Aple-opnGx3c:0.015802)
[&ufboot=100]:0.006692)[&ufboot=99]:0.135986)[&ufboot=99]:0.029913,
((Pmag-opnGx3a:0.183684,Pmag-opnGx3b:0.031645)[&ufboot=100]:0.20812,
('Cfar.opnGx.B1.PE.4':0.046234,(Pyes-opnGx3c:0.085565,Mvar-
opnGx3c:0.098872)[&ufboot=91]:0.010197)[&ufboot=99]:0.110309)
[&ufboot=98]:0.036575)[&ufboot=99]:0.576384)[&ufboot=100]:1.179241)
[&ufboot=100]:0.66963)[&ufboot=100]:0.404268)[&ufboot=99]:0.297195)
[&ufboot=95]:0.264351)[&ufboot=100]:0.630011,((((Mcal-000020:0.135902,
('Medu.opnGx.B2.MY.1':0.035588,Mgal-opnGx10:0.022767)
[&ufboot=100]:0.071943)[&ufboot=100]:1.618933,(Azeb-opnGx7:0.830112,
(Tgra-opnGx10:0.25512,(Sbro-opnGx10:0.018482,Skag-opnGx10:0.049749)
[&ufboot=100]:0.162757)[&ufboot=100]:0.737753)[&ufboot=100]:0.673749)
[&ufboot=69]:0.14624,(Cale-opnGx2:1.888265,(((Pmag-opnGx4a:0.38006,
(Mvar-opnGx4a:0.337291,('Cfar.opnGx.B2.PE.1':0.133006,Pyes-
_opnGx4a:0.095064)[&ufboot=100]:0.199446)[&ufboot=100]:0.17757)
[&ufboot=93]:0.133786,(Ajap-opnGx4a:0.39921,((Airr-
opnGx4a:0.156977,Apur-opnGx4a:0.156121)[&ufboot=100]:0.373184,
('Pmax.opnGx.B2.PE.1':0.154644,Aple-opnGx4a:0.278407)
[&ufboot=100]:0.060526)[&ufboot=100]:0.206762)[&ufboot=100]:0.093705)
[&ufboot=100]:0.878187,(Pmag-opnGx4b:0.518395,((Mvar-opnGx4b:0.371471,
('Cfar.opnGx.B2.PE.2':0.275499,Pyes-opnGx4b:0.098789)
[&ufboot=100]:0.252794)[&ufboot=99]:0.199496,(Ajap-opnGx4b:0.677643,
((Airr-opnGx4b:0.223036,Apur-opnGx4b:0.080473)[&ufboot=100]:0.492104,
('Pmax.opnGx.B2.PE.2':0.227674,Aple-opnGx4b:0.324991)
[&ufboot=100]:0.211031)[&ufboot=100]:0.167918)[&ufboot=100]:0.16221)
[&ufboot=99]:0.160362)[&ufboot=100]:0.999335)[&ufboot=100]:0.28773)
[&ufboot=97]:0.164634)[&ufboot=98]:0.283653,((((Azeb-
opnGx4:2.119158,Azeb-opnGx16:2.367918)[&ufboot=100]:0.23546,(Azeb-
opnGx12:1.289954,(Tgra-opnGx4:0.306904,(Sbro-opnGx4:0.023438,Skag-
opnGx4:0.021489)[&ufboot=100]:0.239761)[&ufboot=100]:1.098656)
[&ufboot=100]:0.51879)[&ufboot=99]:0.16421,(((Azeb-opnGx5:0.166246,
(Azeb-opnGx10:0.158358,Azeb-opnGx11:3.0E-6)[&ufboot=100]:0.17257)
[&ufboot=100]:1.952179,(Azeb-opnGx14:0.857356,(Tgra-opnGx-n2:0.399722,
(Sbro-opnGx12:0.031903,Skag-opnGx12:0.068632)[&ufboot=100]:0.567109)

[&ufboot=100]:1.732517)[&ufboot=100]:1.172291)[&ufboot=75]:0.264968,
((Sbro-opnGx5:0.799989,(Azeb-opnGx6:0.080176,Azeb-opnGx13:0.058699)
[&ufboot=100]:0.618476)[&ufboot=100]:0.688139,((Azeb-opnGx8:1.045823,
(Tgra-opnGx8:0.308682,(Sbro-opnGx8:0.037705,(Skag-
opnGx8a:0.019914,Skag-opnGx8b:0.0154)[&ufboot=100]:0.019024)
[&ufboot=100]:0.19962)[&ufboot=100]:1.039568)[&ufboot=100]:0.745494,
(Azeb-opnGx15:0.716912,((Tgra-opnGx3:0.117517,(Sbro-
opnGx3:0.027255,Skag-opnGx3:0.208065)[&ufboot=100]:0.099721)
[&ufboot=100]:0.292716,(Skag-opnGx6:0.034985,(Sbro-opnGx6:1.0E-6,Sbro-
opnGx7:0.064146)[&ufboot=100]:0.027235)[&ufboot=100]:1.091765)
[&ufboot=100]:0.24981)[&ufboot=100]:0.632278)[&ufboot=99]:0.176534)
[&ufboot=98]:0.209841)[&ufboot=100]:0.396632)[&ufboot=100]:0.209153,
(((Cvir-opnGx5:0.629458,(Cari-opnGx4:0.127793,
('Cgig.opnGx.B2.OS.1':1.0E-6,'Cang.opnGx.B2.OS.1':1.0E-6)
[&ufboot=100]:0.10383)[&ufboot=100]:0.483794)[&ufboot=99]:0.16539,
(Oedu-opnGx4:1.410593,(Cvir-opnGx4:0.26511,(Cari-opnGx5:0.088237,
('Cgig.opnGx.B2.OS.2':0.035211,'Cang.opnGx.B2.OS.2':0.011346)
[&ufboot=100]:0.040877)[&ufboot=100]:0.283801)[&ufboot=100]:0.289632)
[&ufboot=100]:0.421908)[&ufboot=100]:1.689255,((Pvir-opnGx7:0.900883,
((Mcal-000014:0.06556,('Medu.opnGx.B2.MY.2a':0.065146,Mgal-
opnGx3:0.043304)[&ufboot=100]:0.091441)[&ufboot=100]:0.278092,
(Mcal-000015:0.150501,('Medu.opnGx.B2.MY.2b':0.003199,Mgal-
opnGx4:0.005917)[&ufboot=100]:0.162888)[&ufboot=100]:0.415255)
[&ufboot=100]:0.453746)[&ufboot=100]:1.004512,(Pvir-opnGx8:1.080521,
((('Mcal_opsin-052.t1|
Xenopsin':0.093851,'Medu.opnGx.B2.MY.3a':0.385858)
[&ufboot=100]:0.508284,('Medu.opnGx.B2.MY.3b':0.041505,Mgal-
opnGx5:0.093635)[&ufboot=100]:0.390876)[&ufboot=100]:0.343124,
(((Mcal-000017:0.093046,'Myco.opnGx.B2.MY.3a':0.257428)
[&ufboot=100]:0.052598,('Medu.opnGx.B2.MY.3c':0.066567,Mgal-
opnGx6:0.084454)[&ufboot=100]:0.087465)[&ufboot=100]:0.303285,
('Myco.opnGx.B2.MY.3b':0.507579,(('Mcal_opsin-010.t1|
Xenopsin':0.049198,'Myco.opnGx.B2.MY.3c':0.082552)
[&ufboot=100]:0.447827,(('Myco.opnGx.B2.MY.3d':0.379069,
('Medu.opnGx.B2.MY.3d':0.025442,Mgal-opnGx8:0.045936)
[&ufboot=100]:0.306066)[&ufboot=52]:0.035185,(('Mcal_opsin-085.t1|
Xenopsin':0.101905,'Medu.opnGx.B2.MY.3e':0.39063)
[&ufboot=100]:0.305937,('Mcal_opsin-198.t1|Xenopsin':0.073936,
('Medu.opnGx.B2.MY.3f':0.024966,'Mgal_opsin-324.t1|Xenopsin':0.025012)
[&ufboot=100]:0.11612)[&ufboot=100]:0.214563)[&ufboot=99]:0.137831)
[&ufboot=71]:0.098985)[&ufboot=100]:0.100374)[&ufboot=99]:0.123866)
[&ufboot=100]:0.420724)[&ufboot=96]:0.218944)[&ufboot=100]:0.714831)
[&ufboot=100]:0.938125)[&ufboot=98]:0.41011)[&ufboot=77]:0.105379)
[&ufboot=72]:0.145424)[&ufboot=100]:0.244652)[&ufboot=100]:0.418565)
[&ufboot=98]:0.178521)[&ufboot=100]:0.797028)[&ufboot=98]:0.29189)
[&ufboot=100]:1.627729);
end;
