# Supplementary figures and images for: Opsin expression varies across larval development and taxa in pteriomorphian bivalves
Source: Front Neurosci. 2024 Mar 18;18:1357873. doi: 10.3389/fnins.2024.1357873 (PMC10982516; doi:10.3389/fnins.2024.1357873)

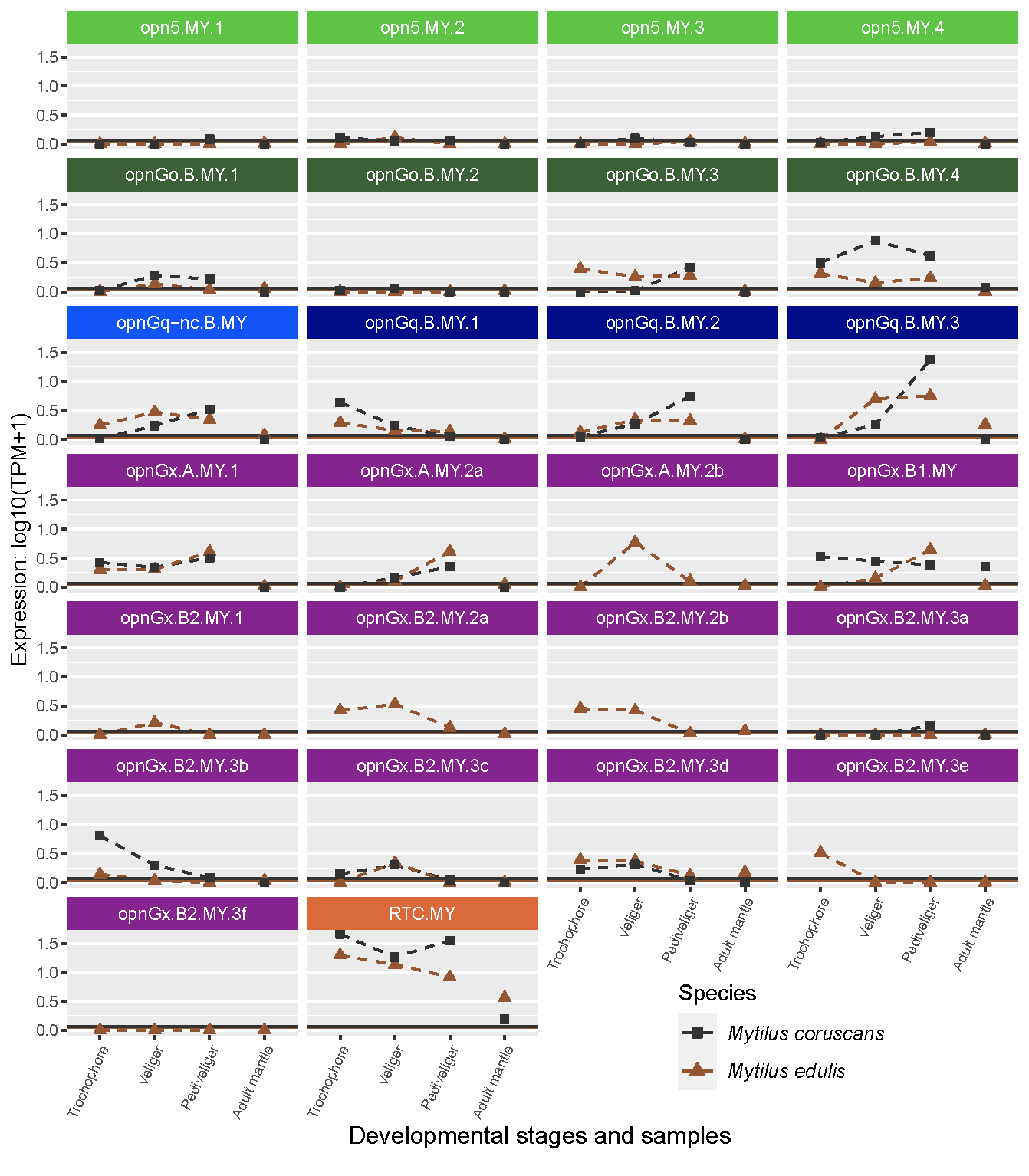

Supplement: Supplementary file 3 [file Image_1.TIFF]

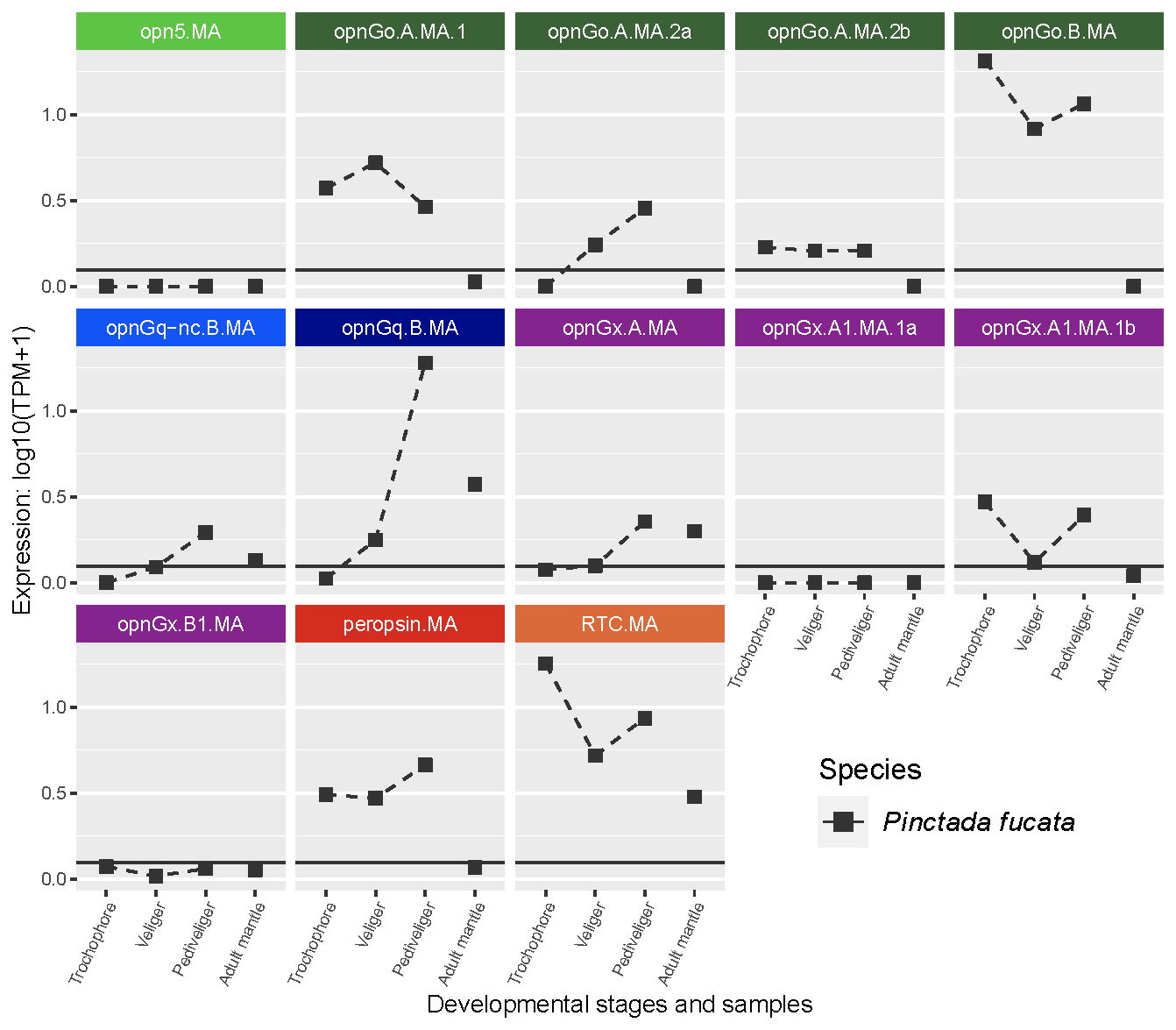

Supplement: Supplementary file 4 [file Image_2.TIFF]
