# Supplementary material for: Opsin expression varies across larval development and taxa in pteriomorphian bivalves
Source: Front Neurosci. 2024 Mar 18;18:1357873. doi: 10.3389/fnins.2024.1357873 (PMC10982516; doi:10.3389/fnins.2024.1357873)
Supplement: Supplementary file 5 [file Table_1.DOCX]

**Supplementary Table S1:** Sequence Read Archive (SRA) metadata for the seven pterimorphian focal species.

|  | **Species** | **Developmental Stage^1^** | **SRA** | **Reads of Sequencing (Million)** | **Bioproject Accession Number^2^** |
| --- | --- | --- | --- | --- | --- |
| **** | ***Mytilus edulis*** | trochophore | SRR6873087 | 30.8 | PRJNA439300 |
|  |  | veliger | SRR6873088 | 37.7 |  |
|  |  | pediveliger | SRR6873089 | 46 |  |
|  |  | adult mantle 1 | SRR8668942 | 40.3 | PRJNA525607 |
|  |  | adult mantle 2 | SRR8668943 | 34.8 |  |
|  | ***Mytilus coruscus*** | trochophore 1 | SRR13364386 | 21.2 | PRJNA689255 |
|  |  | trochophore 2 | SRR13364385 | 25.8 |  |
|  |  | trochophore 3 | SRR13364374 | 23.7 |  |
|  |  | trochophore 4 | SRR13364373 | 27.7 |  |
|  |  | veliger 1 | SRR13364372 | 26.5 |  |
|  |  | veliger 2 | SRR13364371 | 27 |  |
|  |  | veliger 3 | SRR13364370 | 26.1 |  |
|  |  | veliger 4 | SRR13364369 | 29.1 |  |
|  |  | pediveliger 1 | SRR13364382 | 24.3 |  |
|  |  | pediveliger 2 | SRR13364381 | 27.9 |  |
|  |  | pediveliger 3 | SRR13364380 | 25.9 |  |
|  |  | pediveliger 4 | SRR13364379 | 27.9 |  |
|  |  | adult mantle | SRR9090063 | 18.5 | PRJNA543748 |
| **** | ***Crassostrea gigas*** | trochophore 1 | SRR2601701 | 32.1 | PRJNA298285 |
|  |  | trochophore 2 | SRR2601703 | 23.6 |  |
|  |  | veliger 1 | SRR2601698 | 24.7 |  |
|  |  | veliger 2 | SRR2601707 | 21 |  |
|  |  | pediveliger 1 | SRR2601666 | 25.3 |  |
|  |  | pediveliger_2 | SRR2601716 | 20.4 |  |
|  |  | adult mantle 1 | SRR22387571 | 40.6 | PRJNA904561 |
|  |  | adult mantle 2 | SRR22387572 | 44.4 |  |
|  | ***Crassostrea angulata*** | trochophore 1 (1dpf) | SRR14269655 | 28.7 | PRJNA668688 |
|  |  | trochophore 2 (1dpf) | SRR14269654 | 24.3 |  |
|  |  | trochophore 3 (1dpf) | SRR14269647 | 21.8 |  |
|  |  | veliger 1 (4dpf) | SRR14269646 | 27.3 |  |
|  |  | veliger 2 (4dpf) | SRR14269645 | 28.3 |  |
|  |  | veliger 3 (4dpf) | SRR14269644 | 27.3 |  |
|  |  | veliger 1 (7dpf) | SRR14269643 | 28.8 |  |
|  |  | veliger 2 (7dpf) | SRR14269642 | 27.7 |  |
|  |  | veliger 3 (7dpf) | SRR14269641 | 31 |  |
|  |  | pediveliger 1 (17dpf) | SRR14269640 | 27.5 |  |
|  |  | pediveliger 2 (17dpf) | SRR14269653 | 25.5 |  |
|  |  | pediveliger 3 (17dpf) | SRR14269652 | 23.3 |  |
|  |  | pediveliger 1 (21dpf) | SRR14269651 | 26.2 |  |
|  |  | pediveliger 2 (21dpf) | SRR14269650 | 25.4 |  |
|  |  | pediveliger 3 (21dpf) | SRR14269649 | 26.4 |  |
| **** | ***Pinctada fucata*** | trochophore | DRR350405 | 23.1 | PRJDB13068 |
|  |  | veliger | DRR350406 | 23.2 |  |
|  |  | pediveliger | DRR350407 | 22 |  |
|  |  | adult mantle | SRR17133681 | 31.8 | PRJNA786017 |
| **** | ***Chlamys farreri*** | trochophore | SRR5194521 | 13.9 | PRJNA185465 |
|  |  | d-stage veliger | SRR5194520 | 19.2 |  |
|  |  | pediveliger | SRR5194516 | 11.7 |  |
|  |  | adult mantle 1 | SRR5130862 | 25.7 |  |
|  |  | adult mantle 2 | SRR5130868 | 13.1 |  |
|  |  | adult mantle 3 | SRR5130890 | 14.1 |  |
|  |  | adult eye 1 | SRR5130871 | 17.8 |  |
|  |  | adult eye 2 | SRR5130884 | 17.6 |  |
|  |  | adult eye 3 | SRR5130889 | 12.3 |  |
|  | ***Pecten maximus*** | trochophore 1 | SRR2601059 | 31.3 | PRJNA298284 |
|  |  | trochophore 2 | SRR2601062 | 31 |  |
|  |  | veliger 1 | SRR2601057 | 31.7 |  |
|  |  | veliger 2 | SRR2601067 | 23.8 |  |
|  |  | pediveliger 1 | SRR2601049 | 34.9 |  |
|  |  | pediveliger 2 | SRR2601077 | 39.4 |  |
|  |  | adult mantle 1 | SRR14339748 | 22 | PRJNA719586 |
|  |  | adult mantle 2 | SRR14339747 | 20.8 |  |
|  |  | adult mantle 3 | SRR14339746 | 20.5 |  |

^1^ dpf - days post-fertilization

^2^ Deposited at the National Center for Biotechnology Information (NCBI)
