# Supplementary material for: Opsin expression varies across larval development and taxa in pteriomorphian bivalves
Source: Front Neurosci. 2024 Mar 18;18:1357873. doi: 10.3389/fnins.2024.1357873 (PMC10982516; doi:10.3389/fnins.2024.1357873)
Supplement: Supplementary file 6 [file Table_2.DOCX]

**Supplementary Table S2:** Pteriomorphian genome assemblies used mined for opsins. Seven species in bold used in expression analysis (see Supplementary Table S1).

| **Taxonomic Information** | | | **Assembly Source** | **Assembly Stats** | | |
| --- | --- | --- | --- | --- | --- | --- |
| Species | Family | Code | NCBI Accession # or reference | Scaffolds | Length(Mb) | L/N50(Mb) |
| *Scapharca broughtonii* | Arcidae | Sbro | [GigaDB doi http://dx.doi.org/10.5524/100607](http://dx.doi.org/10.5524/100607) | 1026 | 884.57 | 9/44.99 |
| *Scapharca kagoshimensis* | Arcidae | Skag | GCA_021292105.1_ASM2129210v1 | 36 | 1115.24 | 9/60.63 |
| *Tegillarca granosa* | Arcidae | Tgra | GCA_013375625.1 (ASM1337562v1) | 269 | 797.65 | 9/42.62 |
| ***Pinctada fucata*** | **Margaritidae** | **Pfuc** | **GCA_028253585.1 (Pfu_4.1B)** | **29306** | **817.71** | **1343/16.73** |
| *Mytilus californicus* | Mytilidae | Mcal | GCA_021869535.1 (xbMytCali1.0.p) | 176 | 1651.97 | 7/117.87 |
| ***Mytilus coruscus*** | **Mytilidae** | **Mcor** | **GCA_017311375.1 (Mcoruscus_HiC)** | **4434** | **1566.53** | **7/99.54** |
| ***Mytilus edulis*** | **Mytilidae** | **Medu** | **GCA_905397895.1 (MEDL1)** | **3339** | **1827.08** | **465/1.10** |
| *Mytilus galloprovincialis* | Mytilidae | Mgal | GCA_900618805.1 (MGAL_10) | 10577 | 1282.21 | 1904/207.64 |
| *Perna viridis* | Mytilidae | Pvir | GCA_018327765.1 (Pvar_1.0) | 15933 | 731.87 | 49/4.11 |
| ***Crassostrea angulata*** | **Ostreidae** | **Cang** | **GCA_025612915.2 (ASM2561291v2)** | **412** | **624.35** | **5/60.48** |
| *Crassostrea ariakensis* | Ostreidae | Cari | GCA_020458035.1 (ASM2045803v1) | 60 | 663.15 | 5/66.34 |
| ***Crassostrea gigas*** | **Ostreidae** | **Cgig** | **GCA_902806645.1 (cgigas_uk_roslin_v1)** | **236** | **647.89** | **5/58.46** |
| *Crassostrea virginica* | Ostreidae | Cvir | GCA_002022765.4 (C_virginica-3.0) | 10 | 684.72 | 4/75.94 |
| *Ostrea edulis* | Ostreidae | Oedu | GCA_947568905.1 (xbOstEdul1.1) | 1364 | 935.15 | 5/95.56 |
| *Amusium pleuronectes* | Pectinidae | Aple | https://doi.org/10.1038/s41559-022-01898-6 | 310 | 669.66 | 9/35.37 |
| *Argopecten irradians* | Pectinidae | Airr | <https://doi.org/10.1038/s41559-022-01898-6> | 815 | 882.75 | 87/3.14 |
| *Argopecten purpuratus* | Pectinidae | Apur | <https://doi.org/10.1038/s41559-022-01898-6> | 315 | 781.61 | 41/5.53 |
| ***Chlamys farreri*** | **Pectinidae** | **Cfar** | [**https://doi.org/10.1038/s41559-022-01898-6**](https://doi.org/10.1038/s41559-022-01898-6) | **102** | **966.73** | **9/50.08** |
| *Mimachlamys varia* | Pectinidae | Mvar | GCA_947623455.1 (xbMimVari1.1) | 83 | 975.38 | 9/50.75 |
| *Mizuhopecten yessoensis* | Pectinidae | Pyes | <https://doi.org/10.1038/s41559-022-01898-6> | 828 | 1171.90 | 9/60.01 |
| ***Pecten maximus*** | **Pectinidae** | **Pmax** | **GCA_902652985.1 (xPecMax1.1)** | **3983** | **918.31** | **10/44.82** |
| *Placopecten magellanicus* | Pectinidae | Pmag | <https://doi.org/10.1038/s41559-022-01898-6> | 4054 | 1458.27 | 10/66.32 |
| *Ylistrum japonicum* | Pectinidae | Ajap | <https://doi.org/10.1038/s41559-022-01898-6> | 31 | 695.21 | 8/39.05 |
