# Supplementary material for: Opsin expression varies across larval development and taxa in pteriomorphian bivalves
Source: Front Neurosci. 2024 Mar 18;18:1357873. doi: 10.3389/fnins.2024.1357873 (PMC10982516; doi:10.3389/fnins.2024.1357873)
Supplement: Supplementary file 7 [file Table_3.DOCX]

**Supplementary Table S3:** Dataset-specific threshold values of transcripts per million (TPM) for the seven pteriomorphian focal species.

| **Family** | **Species** | **10% cut-off value** | |
| --- | --- | --- | --- |
| Mytilidae | *Mytilus edulis* | 0.125737 |  |
|  | *Mytilus coruscus* | 0.158149 |  |
| Ostreidae | *Crassostrea gigas* | 0.294612 |  |
|  | *Crassostrea angulata* | 0.100678 |  |
| Margaritidae | *Pinctada fucata* | 0.247975 |  |
| Pectinidae | *Pecten maximus* | 0.171384 |  |
|  | *Chlamys farreri* | 0.235579 |  |
